# Initial Coin Offerings

**DOI:** 10.1371/journal.pone.0233018

**Published:** 2020-05-21

**Authors:** Paul P. Momtaz

**Affiliations:** UCLA Anderson School of Management, Los Angeles, California, United States of America; National Institute of Public Finance and Policy, INDIA

## Abstract

This paper examines the *market for initial coin offerings* (*ICOs*). ICOs are smart contracts based on blockchain technology that are designed for entrepreneurs to raise external finance by issuing tokens without an intermediary. Unlike existing mechanisms for early-stage finance, tokens potentially provide investors with rapid opportunities thanks to liquid trading platforms. The marketability of tokens offers novel insights into entrepreneurial finance, which I explore in this paper. First, I document that investors earn on average 8.2% on the first day of trading. However, about 40% of all ICOs destroy investor value on the first day of trading. Second, I explore the determinants of market outcomes and find that management quality and the ICO profile are positively correlated with the funding amount and returns, whereas highly visionary projects have a negative effect. Among the 21% of all tokens that get delisted from a major exchange platform, highly visionary projects are more likely to fail, which investors anticipate. Third, I explore the sensitivity of the ICO market to adverse industry events such as China’s ban of ICOs, the hack of leading ledgers, and the marketing ban on FaceBook. I find that the ICO market is highly susceptible to such environmental shocks, resulting in substantial welfare losses for investors.

Initial Coin Offerings (ICOs) or token sales are smart contracts based on distributed ledger technology (DLT or blockchain) designed to raise external finance by issuing *coins* or *tokens*. Smart contracts are computer protocols that automatize value-exchange transactions between the entrepreneur and investors, potentially creating perfect disintermediation. So far, until the end of 2019, over 5,600 ICOs have raised more than USD 27 billion (retrieved from https://icobench.com/ on January 16, 2020). From an entrepreneur’s perspective, ICOs are attractive as they offer funding at all stages with global investor outreach at close-to-zero transaction costs, although entrepreneurial firms dominate the pool of ICO firms thus far. From an investor’s perspective, ICOs are attractive as they potentially offer more rapid exit options thanks to liquid token exchanges. However, there is a regulatory distinction between utility, security, and cryptocurrency tokens (see, for a detailed discussion, Momtaz [1] and Section I in this article). While the latter two token types fall under securities or asset laws, *utility tokens* operate in a legal grey zone. Utility tokens essentially charter a promise that the token can be redeemed for the ICO project’s products or services once they are developed. But investors in utility tokens currently do not hold enforceable claims in many jurisdictions, which seems to be in conflict with the corporate governance and law and finance literature [2, 3]. Therefore, the purpose of this study is to provide an empirical characterization of the ICO market.

This study contributes to an emerging body of contemporaneous research on ICOs. Theoretical work is diverse and presents a dynamic asset-pricing model for tokens [4], a model of token value from a consumer demand perspective [5], a model of tokens as membership in peer-to-peer platforms and compensation for miners [6], an agency theory comparing the optimality of ICOs to more traditional venture capital [7], a model rationalizing ICOs for building peer-to-peer platforms [8], and a theory of optimal token contract design [9].

Empirical work examines general ICO success along various dimensions [10, 11, 12] as well as the determinants such as an agency-related explanation [13], the price difference between the ICO and the first trading price [14], the long-run performance of ICOs and token volatility [15, 16], token liquidity [17], investor sentiment and the timing of ICOs [18], the role of information disclosure and signaling for ICO success [19, 20, 21, 22], a moral hazard-based explanation of ICO market outcomes [23], a wisdom of the crowd-related test of ICO success [24], the role of large and institutional investors [25, 26, 27] and aggregator platforms [28], as well as the geographic determinants of ICOs [29].

Although the empirical work is rapidly evolving and has produced important insights into the functioning of the ICO market, a comprehensive empirical characterization of ICOs is still missing. (I acknowledge, however, that there are concurrent efforts toward a comprehensive characterization of the ICO market (see, for example, Lyandres, Palazzo, and Rabetti [16]. and Howell, Niessner, and Yermack [17]). Block et al. [30] compare crowdfunding to ICOs and Kher, Terjesen, and C. Liu [31] provide a broader review of the blockchain, cryptocurrency, and ICO literature.) This paper aims to fill this gap. My paper is closely related to concurrent work by Kostovetsky and Benedetti [14] and Howell, Niessner, and Yermack [17]. Kostovetsky and Benedetti [14] examine the determinants of ICO underpricing. In keeping with the IPO literature, they define underpricing as the relative difference between issuance and opening prices. In contrast, I examine first-day returns, defined as the relative difference between opening and closing prices. The definition is also used in the IPO context (see, e.g., [32]). Both measures reflect the financial incentives provided to potential investors by the ICO firm, but at different points in time. Kostovetsky and Benedetti’s [14] measure reflects investors’ incentive to invest in the ICO at all, while my measure reflets their incentive to create a liquid after market. Both measures are important as they reflect different aspects of the ICO market. Howell, Niessner, and Yermack [17] study, inter alia, the determinants of ICO firms’ operating success (in terms of the number of employees) and the probability of exchange listings. My work sheds light on similar aspects of operating success, namely, the time it takes ICO firms to successfully complete their fundraising campaign. Moreover, I complement Howell, Niessner, and Yermack’s [17] evidence on the listing decisions with analyses of the time-to-listing and the probability of delistings, which are aspects not covered in prior work.

The paper is structured in four parts. First, it gives a comprehensive conceptual overview over the ICO phenomenon, covering token types, the life cycle of a typical ICO, a discussion of key advantages and challenges, and a detailed comparison between ICOs and more conventional financing methods. Second, it provides extensive descriptive statistics, covering relative (in%) and nominal (in US$) first-day returns, gross proceeds, time-to-market, and project failure. Third, it explores potential determinants of these ICO market characteristics. Fourth, it sheds light on the market effects of adverse industry events such as regulatory bans and technical vulnerabilities.

My empirical results indicate that ICOs create, on average, investor value in the short run. The first-day mean returns, measured as raw and as equally- and value-weighted abnormal returns, range from 6.8% to 8.2%. The range is significantly higher than that for median first-day returns, which lies between 2.6% and 3.4%. In fact, between 39.5% and 45.7% of all ICOs result in negative first-day returns and hence destroy investor value. The average magnitude of first-day returns does not significantly change over the sample period (2015–2018). Overall, these estimates are clearly below the first-day returns for IPOs during the dot-com bubble that averaged at about 40% [32].

As for the other ICO market characteristics, the distribution of ICO gross proceeds is positively skewed with mean $15.1 million and median $5.8 million. This reflects the fact that most funding is concentrated around a small number of ICOs. 37% of the total funding raised in 2017 was made by only 20 ICOs (for details, see [1]; [33]). The amount of ICO gross proceeds is significantly increasing over time. Over the sample period, average gross proceeds increase by $13,000 per day. These findings add to Catalini and Gans [5], who show that ICO funding is higher when the amount of token supply is limited. Furthermore, average nominal first-day returns, calculated as the first-day raw return multiplied with the ICO gross proceeds, is $1.1 million, though the median is zero.

Turning to time-to-market indicators, the average (median) time from project initiation, as reported by the firms themselves, to the ICO start is 598 (312) days. After the ICO, it takes the average (median) firm another 93 (42) days to get listed on a token exchange platform. Interestingly, 21% of the projects get delisted subsequently from at least one of the major exchange platforms, while 12.9% get delisted from every major platform, which is effectively a project’s death. Note that I focus on the 26 major platforms that were tracked on Coinmarketcap, although about 200 exchanges existed during the sample period. This does not seem to be an issue because a delisting from all major exchanges usually causes token prices to fall to zero.

Next, a regression framework is presented to shed more light on the the determinants of these ICO market characteristics. In line with existing research in entrepreneurial finance [34, 35], I assume that investment decisions are heavily based on the anticipated project quality as a reference point and derive a number of testable hypotheses related to the following three proxies for project quality: quality of the management team, platform vision, and ICO profile. The hypotheses predict that, generically speaking, the ICO success is positively affected by the quality of the management team and the project’s ICO profile, while acknowledging that a prediction about the project’s vision is ambiguous due in part to the fact that visionary projects are often less likely to be implemented.

The regression results of first-day returns on the three proxies for project quality and a vector of other explanatory variables confirm my empirical predictions. In particular, the quality of the management team is significantly positively related to first-day returns (as is the ICO profile, albeit insignificantly). Interestingly, the project’s vision has a detrimental effect on first-day returns. A subsequent analysis shows that this can be explained by the fact that highly visionary projects are more likely to fail. Furthermore, the results suggest that general crypto-market sentiment and whether the project uses a standardized technical process to conduct its ICO (*ERC20*, see section I) also positively affects first-day returns. Moreover, these results hold when first-day returns are replaced as the dependent variable by an indicator variable of positive first-day returns, suggesting that extreme outliers are not driving the results.

The analysis of the determinants of ICO gross proceeds and nominal first-day returns suggests that, in keeping with the above, the quality of the management team and the project’s ICO profile has a positive effect, while the project’s vision reduces both amounts. However, only the coefficient on ICO profile is highly significant in the gross-proceeds regressions. A one standard deviation increase in ICO profile is associated with an increase in gross proceeds of $2.44 million. Moreover, ICO gross proceeds are lower when a project conducts a *Pre-ICO* and decrease with the duration of the actual ICO, while they are increasing in market sentiment and when projects accept legal tender as means of exchange for tokens. Nominal first-day returns are negatively affected by the project’s vision, which is consistent with the finding that highly visionary projects are more likely to fail and result therefore in lower first-day returns. Nominal first-day returns decrease also when an ICO involves a know-your-customer (KYC) process, in which the project team gathers information from investors to be compliant with anti-money laundering laws. Finally, ICO size and country restrictions increase nominal first-day returns, with the latter implying that projects have to create stronger incentives to attract investors if they restrict the pool of potential investors.

In addition, this study provides evidence on the determinants of time-to-market indicators and project failure. Time-to-market is reduced by a professional ICO profile, but delayed if the project uses a KYC process and accepts legal tender in exchange for its tokens. Project failure can be predicted fairly accurately using the three proxies for project quality. A one standard deviation increase in the quality of the management team reduces the probability of project failure by 19.8%. Similarly, a one standard deviation increase in the project’s vision increases the probability of project failure by 21.5%. This finding gives an important explanation for why investors are reserved when facing promising project visions. Further, ICO profile has an economically weak but statistically significant effect on project failure.

The final section of the paper sheds some light on the sensitivity of the ICO market to adverse industry events. In particular, a regression framework is employed to analyze the largest hacks of cryptocurrency projects, the most severe regulatory bans by the Chinese and the South Korean governments, and the recent Facebook announcement to ban ICO ads. These drastic events had a dramatic market impact and spurred much debate. The events are explained in detail in section VII. I construct an aggregate index for ICOs taking place within one month after the focal event. First-day returns are regressed on the index and on the events separately. The results are statistically and economically significant. On average, the first-day returns diminish after the events, using the aggregate-index model. The coefficient is -7.62%, which compares in magnitude to the average first-day returns of 6.8% to 8.2%. When I test for the events’ effects separately, events casting doubt on the technical underpinnings of the projects (and the entire industry) entail significantly worse market reactions than governmental interventions. For example, the hack of *Parity Wallet*, a leading digital wallet service provider that is linked to the *Ethereum blockchain*, resulted in a decline in first-day mean returns of 16.93%. This suggests that the hack reversed the positive average first-day returns into wealth losses for investors. In contrast, the Chinese ban of ICOs together with declaring ICOs an illegal activity lead to an average decrease of first-day mean returns of 6.01%. Similarly, the South Korean ICO ban is associated with an average decrease of 5.76%.

This study makes at least two contributions to the emerging literature on ICOs. First, it provides comprehensive empirical evidence of ICO market characteristics and determinants, complementing concurrent papers such as Howell, Niessner, and Yermack [17], Kostovetsky and Benedetti [14], and Lyandres, Palazzo, and Rabetti [16] as well as an accessible conceptual overview over the life cycle of ICOs, token types, advantages and challenges, and features distinguishing ICOs from other forms of external finance. The descriptive statistics show there is considerable skewness in all dimensions in the ICO market, an important feature which has to be accounted for in theoretical work. First-day returns, gross proceeds, and time-to-market are all positively skewed. Average first-day returns are positive for the mean and the median firm. There are competing explanations for the observed level of first-day returns. One explanation is that token issuers have an incentive to set the opening price below the expected equilibrium price in order to generate market liquidity as a knock-on effect for platform growth, which, in turn, may increase the inherent token value [36]. Another explanation might be that the sample captures a ‘hot market’ in which investors overbid when tokens start trading [37, 38]. It is left to future research to disentangle the possible competing explanations. Either way, both explanations suggest positive first-day returns, which is consistent with the empirical evidence. Moreover, examining a comprehensive set of ICO market characteristics, the paper is able to distinguish determinants that are consistent across all characteristics from those that only predict certain market characteristics. Specifically, it seems that the measures related to the quality of the management team, the ICO profile, and the project’s vision seem reliable predictors of ICO success. All three measures are determined by a large number of industry experts, suggesting that the *wisdom of the crowd* works effectively in the ICO market.

A second contribution relates to the study of regulatory events, technical vulnerabilities, and the *FaceBook* ban. The ICO market reacted highly sensitively to all three event types, although the magnitude of how the event types affected tokenholders differ. Regulatory bans of ICOs in China and South Korea wiped out initial gains to investors worldwide, whereas technical hacks even imposed significant losses onto holders of unrelated tokens. In fact, the findings suggest that more than twice as much market uncertainty stems from technical issues compared to regulatory actions. The results help explain the high observed volatility in token prices [16], [15], [39]. The analysis has implications for theoretical work guiding policy-making (e.g., [8], [4], [40].

The remainder is organized as follows: Section I provides some background on ICOs, testable hypotheses are developed in section II, and section III presents the data and initial results. The regression results are discussed in sections IV (first-day returns), V (gross proceeds and nominal first-day returns), VI (time-to-market and project failure), and VII (sensitivity analysis of industry events). Section VIII discusses important limitations of my study and section IX concludes.

## I. Initial Coin Offerings: An overview

*Initial Coin Offerings* (*ICOs*) or *token sales* are a mechanism to raise external funding through the emission of tokens. Conceptually, tokens are entries on a *blockchain* (or a *digital ledger*). The blockchain records all transactions made in the cryptocurrency chronologically and publicly. The owner of the token has a *key* that lets her create new entries on the blockchain to re-assign the token ownership to someone else. A useful distinction of token types is the following as it determines the legal status of the token (see, for a more comprehensive overview, Momtaz [1] and Momtaz, Rennertseder, and Schroeder [33]):

1. *Utility tokens*: The most common type of tokens assigns a right to redeem the token for a product or service once developed. There is no ownership right attached to utility tokens. The token type is popular due to the low degree of regulation in most jurisdictions. It is interesting from a research perspective as it unifies a payment and an investment instrument, and is hence the focus of this study.
2. *Security tokens*: The token type usually conveys voting power and is subject to securities laws determined by the *Howey Test* (see below). Until the end of 2018, about 3% of all ICOs involved security tokens.
3. *Cryptocurrency tokens*: The token type is a general-purpose store of value or medium of exchange token. At least for the purpose of taxation, cryptocurrency tokens fall under asset laws in most jurisdictions. The most prominent cryptocurrency token is *Bitcoin*.

The rest of this section provides a comparison of ICOs to conventional financing methods, a discussion of the life cycle of a typical ICO, an overview of the evolution of the ICO market, as well as ICO advantages and challenges.

### A. Comparison of ICOs to conventional financing methods

This section provides a brief comparison of ICOs to conventional financing methods such as reward and equity crowdfunding, venture capital, and initial public offerings (IPOs) along the dimensions start-up or firm characteristics, investor characteristics, deal characteristics, and post-deal characteristics. An overview is provided in Table 1. Other excellent comparisons of ICOs and conventional financing methods are presented in Lipausch [41] and Blaseg [19], on which this section draws to some extent.

**Table 1:** Comparison of Initial Coin Offerings to (Reward and equity) crowdfunding, venture capital, and initial public offerings.

	Initial Coin Offerings	Reward Crowdfunding	Equity Crowdfunding	Venture Capital	Initial Public Offerings
Panel A: Start-up or Firm Characteristics					
Funding stage	Theoretically all stages	Before seed stage (prototype)	Early stage	Balanced-stage	After later stage
Issuance	Utility tokens, cryptocurrencies, or security tokens	Product (vouchers)	Equity-like instruments	Equity shares	Equity shares
Panel B: Investor Characteristics					
Investors	All types	Early adopters	Angel investors	Limited partners	Public
Motivation	Financial and non-financial	Financial and non-financial	Financial and non-financial	Financial	Financial
Panel C: Deal Characteristics					
Investment amounts	>$100k	$1k—$150k	$100k—$2m	$500k—$10m	>$10m
Transaction costs	Low	Low	Low	Medium	High
Information basis	Whitepaper	Project description	Business plan and pitch deck	Business plan and pitch deck	IPO prospectus
Degree of regulation	Low	Low	Low	Medium	High
Panel D: Post-Deal Characteristics					
Liquidity	High (if listed)	Low	Low	Low	High
Voting rights	Security tokens: yes; utility tokens and cryptocurrencies: no	No	No	Yes	Yes
Exit options	ICO, open market	IPO, acquisition	IPO, acquisition	IPO, acquisition	Open market

#### Start-up or firm characteristics

Unlike ICOs, conventional financing methods are tailored to specific funding stages. Crowdfunding is used to fund early stages, venture capital covers all stages (balanced-stage) until a firm goes public, and IPOs are used to acquire high-volume growth capital for established start-ups. ICOs, in contrast, can theoretically be employed during all funding stages, although entrepreneurial firms dominate the pool of firms raising capital through ICOs. In fact, examples of successful ICOs cover funding amounts from about $100,000 up to $4.2 billion (as of July 2018) (for details, see [1]; [33]). Another important distinction is that investors obtain products or equity-like instruments in crowdfunding campaigns, while venture capitalists or IPO investors receive stocks. Again, ICOs are used to issue all this and more, i.e. equity shares (security tokens), products or services (or the rights to buy them once developed) (utility tokens), and mediums of exchange (cryptocurrency tokens).

#### Investor characteristics

In a similar vein, while ICOs are suitable to attract all different kinds of investors (from early adopters over altruistic investors to institutional investors), conventional financing methods usually attract specific types of investors. Reward and equity crowdfunding attracts early adopters and angel investors, respectively. Venture capital and IPOs are traditionally more attractive to sophisticated investors. Further, the motivation of investors differs among these financing methods. Venture capitalists and IPO investors are more likely to be driven by financial motives, while ICO and crowdfunding investors are often equally driven by financial motives and non-financial motives (altruism, product interests, feedback provision, etc.) (see [41]).

#### Deal characteristics

A major reason for the soaring popularity of ICOs is that they have close-to-zero transaction costs and keep documentation needs and regulation similar to crowdfunding campaigns at a minimum, but potentially enable start-ups to raise substantial funding comparable to costly and highly regulated venture capital transactions or IPOs. In fact, looking only at the first half of 2018, the largest ICO ranks in terms of funding amount among the three largest IPOs globally (see [17]). Interestingly, the largest ICO exceeds the aggregate funding raised on the premier crowdfunding platform Kickstarter since its inception in 2009 [22].

#### Post-deal characteristics

A major reason for investors to invest in ICOs is the after-market liquidity. Although not the case for all tokens, many tokens get listed on a token exchange platform, which is open 24/7 for online trading, within three months after the ICO ends. Neither crowdfunding campaigns nor venture capitalists are able to provide similar levels of liquidity. Consistent with liquidity discount theories (e.g., [42]), the liquidity of tokenized start-ups adds value that is shared within the decentralized network. Another notable design advantage of ICOs is that they can flexibly convey voting rights, depending on the token type issued. Finally, perhaps the most striking ICO advantage that boosts rapid innovation is the exit method. Exits in crowdfunding campaigns or venture capital are often not realizable before a certain maturity stage and not realizable in the short-run as a potential acquirer needs to be identified or an IPO needs to be prepared. In contrast, ICOs provide the earliest exit option of all financing methods by delegating the future development of a platform to a decentralized network of developers and supporters often before a product prototype or service is developed. While most ICO projects retain a token share, the liquidity of tokens guarantees prompt exits at any time, provided that the token is listed.

### B. The lifecycle of a cryptocurrency

#### B.1. Project development, marketing, and the Howey Test

In most projects, marketing the project starts almost as early as the project itself. Once the core team has defined its vision, early marketing activities include building a professional website and a heavy use of social media and slack and telegram channels. After all, the value of the new cryptocurrency is closely related to the size of its network. Closer to the ICO (or Pre-ICO), a whitepaper will be published and the core team goes on roadshow to meet with potential investors.

A crucial step in the phase preceding the ICO is the *Howey Test* to ensure that the project’s token does not fall under the legal definition of a security and is hence subject to securities regulation. The Howey Test was developed in a U.S. Supreme Court case in 1946 and lays down criteria according to which a token might be considered a security from a regulatory standpoint. The four main criteria are (i) there is investment of money, (ii) profits are expected, (iii) money investment is a common enterprise, and (iv) any profits come from the efforts of a promoted or third party. The feature that most projects exploit to pass the Howey Test is that they make a decentralized cryptocurrency that is equivalent to a currency (or simply cash) with no central owner.

#### B.2. Pre-ICO

Many projects (about 44% in the sample used in this study) choose to conduct a Pre-ICO. A Pre-ICO usually has a lower desired fundraising amount and provides an incentive to early adopters by issuing the tokens cheaper than in the ICO. The motives for Pre-ICOs are often to cover the costs for the actual ICO such as the costs incurring due to promotional ads, strategic hires, and the roadshow. An interesting feature of Pre-ICOs is that they can be seen as a mechanism to elicit information from potential investors about the fair price of the token and the total funding amount that is possible, which can be used to increase the effectiveness of the actual ICO.

#### B.3. ICO

There is no rule of thumb as to when an ICO takes place and how long it endures. While some ICOs are closed within a day (or even less time), others endure for a year and more. However, there is some movement towards standardization in the ICO market. Most tokens are created on the *Ethereum blockchain*. The technical standard is referred to as *Ethereum Request for Comment 20* (or, in short, *ERC20*), which provides a list of rules that a token built on the Ethereum blockchain has to implement. As of January 2019, more than 165,000 tokens had been created based on ERC20, which corresponds to more than 80% of the market share (the estimate comes from https://etherscan.io/tokens, retrieved January 7, 2019).

The process of creating a token is very straightforward and a token can basically be created within minutes. The code can be downloaded from Ethereum’s website and then easily be manipulated along a dimension of parameters such as the total amount of tokens, how fast a block gets mined, and whether to implement a possibility to freeze the contracts in case of emergency (e.g., a hack). The ease with which tokens can be created thanks to Ethereum was a main driver for the rise in ICOs as it makes creating new cryptocurrencies not only more time-efficient but also less technical.

The mechanics of the actual ICO are almost as easy as sending an email. The project creates an address to which the funds will be sent. The token will then be paired with other currencies (virtual and possibly fiat) that the project accepts as payment for its token. Investors send then funds (only the paired currencies) to the address and receive the equivalent amount of tokens.

#### B.4. Listing

A critical milestone for every cryptocurrency is the listing on a token exchange following the ICO. The listing ensures that the tokens can be traded, hence it provides the main source of liquidity. Liquidity attracts new investors and paves the way for the use of the token as an actual currency.

The requirements for a project to get listed are relatively opaque but seem, in general, not very rigorous. *Poloniex*, a large exchange platform, states: “We don’t have a definitive set of criteria as each project is unique. We listen to the community and select projects that we believe are unique, innovative, and that our users would be interested in trading (the quote comes from https://www.coinist.io/how-to-get-your-digital-token-listed-on-an-exchange/, retrieved December 8, 2018). Another major platform, *Bittrex*, gives more guidance as to what is required to get listed. They require a self-explanatory token name, a description of the project, a trading symbol, a logo, a launch date of the ICO, at least one team member or shareholder (more than 10%) having their identity verified, a Github link to the project’s source code, and a number of rather innocuous information such as the maximum money supply, other exchange listings, how money was raised.

For the majority of the cryptocurrencies, the journey ends with a delisting that is effectively a project’s death as there is no platform for the currency to be exchanged. In February 2018, as many as 46% of 2017’s ICOs had already reportedly failed (for details, see [1]; [33]).

### C. More ICO advantages and some challenges

#### C.1. Advantages

Perhaps the most striking advantage is that the technical flexibility of smart contracts allows this novel mechanism to replace all other financing methods by mimicking their distinct features at close-to-zero transaction costs. Amsden and Schweizer [11] provide an excellent outline of the technical details.

Another major economic benefit is that ICOs lower the commitment requirements to innovate as they help delegate the development of the innovation to a decentralized network and potentially provide the initial innovators with rapid exit options thanks to the liquidity that comes along with token listings on exchange platforms. Anecdotal evidence shows that this mechanism attracts innovators who would otherwise be less likely to become innovators. Examples include *Brendan Eich* who left his appointment as CEO of the *Mozilla Corporation* to found a new browser called *Brave* with ICO proceeds of $35 million, which were raised within only 30 seconds [43]. Another example is *Will McDonough* who left his top-executive position at *Goldman Sachs* to launch an ICO for a blockchain-based firm offering smart contract solutions [44]. Taken together, the anecdotal evidence suggests that ICOs provide a means to innovate that attracts all types of potential innovators.

ICOs are also attractive for innovators because they help gauge consumer demand from future users and the firm’s market value at an early stage [5], [8]. This early signal helps innovators to improve platform features. From the users and investors perspective, ICOs may help redistributing platform gains to platform developer’s and user’s instead of financial intermediaries in most conventional financing methods [17].

Finally, an important advantage is that ICOs align the incentives between developers, users, and miners without the need to give any party more control over the platform. This might spur business models that have previously relied heavily on voluntary work such as *Wikipedia*’s business model based on openly edible content [45]. ICOs can spur such innovations by compensating initiators as well as later contributors.

#### C.2. Challenges

There are a number of risks associated with investing in cryptocurrency projects. While there is the obvious risk of depreciation of the token price that cryptocurrencies have in common with regulated investments (although the volatility of cryptocurrencies is much higher [16], 15]), there are idiosyncratic risks attached to this new asset class.

First, the ICO market has been criticized of providing a fertile soil for *scams*. Indeed, there have been some scams, however, recent research suggests that, using a conservative definition of what constitutes a scam, the number of scams amounts to about 40 cases [46]. In fact, it seems that market participants see through fraudulent behavior. For example, Blaseg [19] shows that a large amount of blockchain-based start-ups is not able to secure funding in ICOs. This observation is backed by a popular database called *Ether Scam Database* that documents questionable activities and warns potential investors (https://etherscamdb.info). Nevertheless, it remains an open issue to what extent betrayed investors can be compensated. One issue is that the blockchain is pseudo-anonymous, meaning that it is difficult to track where embezzled funds go to. Another issue is that ICO projects operate globally, and hence it is unclear whether and how a national enforcer could prosecute fraudulent activity.

Second, *asymmetric information* is a major challenge given the absence of functioning institutions in this infant market. Chod and Lyandres, [7] show that severe information asymmetry might render the ICO market into a ‘market for lemons.’ Empirically, Howell, Niessner, and Yermack [17] attest to the dearth of basic information about the issuer and Momtaz [23] shows that ICO projects have an economic incentive to exacerbate the information asymmetry by exaggerating information disclosed in whitepapers. Closely related, asymmetric information paired with the lack of institutions might result in the occurrence of moral hazard [23].

Third, tokens do not convey voting power to investors, due in large part to the Howey Test. While this may make early projects more agile and flexible, and hence may promote early growth, it is unclear, however, how the lack of influence and corporate governance will affect project value and success as the project matures.

Fourth, network effects might turn out to be a major risk. Despite the fact that cryptocurrencies started out in defiance of the traditional financial system that they wanted to decentralize, the gravitation towards Ethereum to design tokens generates systematic risks.

### D. The evolution of the ICO market

**Fig 1:**
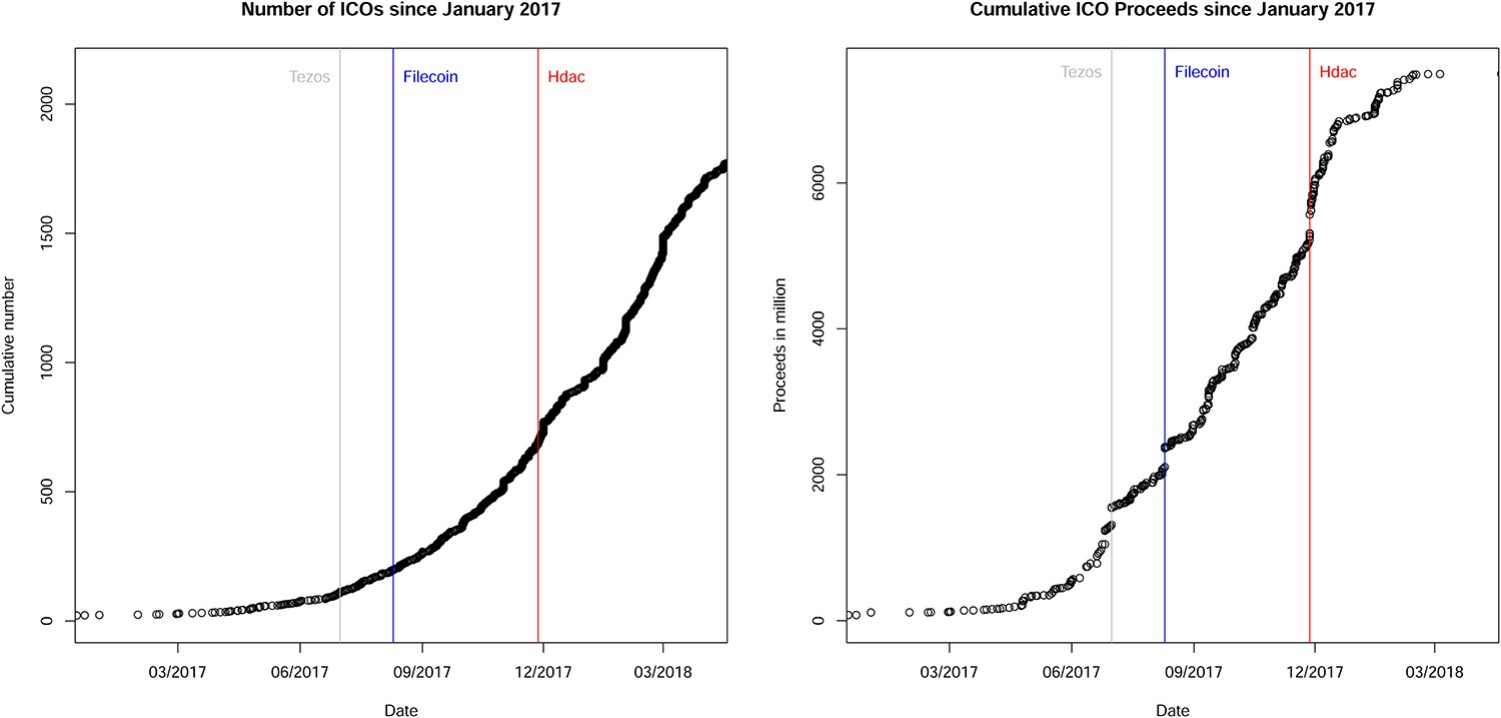
The evolution of the ICO market: Cumulative number of ICOs (lhs) and ICO proceeds (rhs) since January 2017. Three major ICOs in terms of ICO volume during my sample period are shown as vertical lines (Tezos, Filecoin, and Hdac). The total number of ICOs in the sample is 2,131. Thereof, estimates of gross proceeds are available for 501 ICOs.

The first ICO took place in July 2013. The *Mastercoin* project (now *Omni*) was able to raise more than $5 million in Bitcoins. Since then, about 5,000 firms have announced an ICO as of January 2019 and more than 165,000 tokens have been created on the Ethereum blockchain. However, about 37% of the total ICO proceeds in 2017 were made by only 20 ICOs (for details, see [1]; [33]). For a more comprehensive overview of the evolution of the ICO market, I plot the number of ICOs and the volume of ICO proceeds in Fig 1.

## II. Main hypothesis and determinants of ICO success

The overarching conjecture is that ICO projects attract investors by offering substantial short-term financial rewards. Drawing on the IPO literature, there may be several reasons for high initial returns to investors. One explanation is the *market liquidity hypothesis* [36]. ICO projects have an incentive to underprice their tokens to generate market liquidity as a knock-on effect to signal platform growth prospects. Liquidity is important for several reasons. First, unlike other entrepreneurial financing methods, tokens allow entrepreneurial firms to mitigate the illiquidity discount, which can result in raising substantially more growth capital. Second, Trimborn, M. Li, and Härdle [47] show that liquidity can create token demand from a portfolio-choice perspective, which increases token value. Third, in a similar vein, liquidity can increase user adoption of ICO platforms, which increases the ICO platform’s inherent value [17]. Underpricing (or high first-day returns) may hasten these liquidity effects. It rewards early investors for risk-taking and market signaling, attracts new investors, and accelerates these liquidity-based network effects.

There are other potential explanations from the IPO underpricing literature that also suggest positive initial returns in ICOs (see, for an excellent survey, Ljungqvist, [32]). Asymmetric information models of underpricing such as the winner’s curse [48], information revelation [49], and signaling [50, 51, 52] argue that the issuing firm has to offer underpricing to retain the uninformed investors. Institutional theories regards underpricing as *legal insurance* because price discounts reduce the probability of future lawsuits from tokenholders disappointed with the post-ICO performance [53]. Finally, there are behavioral theories such as the investor sentiment and ‘hot market’ explanation that maintains that overly optimistic investors who start investing in the aftermarket bid the token price beyond its true value [37, 38, 54]. Drobetz, Momtaz, and Schröder [18] provide a first analysis of the role of investor sentiment in ICO markets. Given that there are many, sometimes competing, explanations of ICO underpricing and first-day returns, it is left to future research to disentangle the relative merits of each one. Nevertheless, the important point of all potential explanations is that they suggest that initial returns are also positive in the context of ICOs, a key prediction of this study.

Another interesting feature about the ICO phenomenon is the fact that investors invest substantial amounts of money in utility tokens although their investments are neither governed by legal rights nor by firm-level corporate governance. The only reference point for their investment decisions are observable characteristics of the project and even those observable characteristics are quite limited given the young age at which most projects enter the ICO market. However, an industry standard has formed around three indicators on which an expert crowd shares opinions that are common across several platforms on which ICOs are marketed. These indicators are the quality of the management team, the project’s vision, and its ICO profile.A potential issue with these ratings is that expert raters are allowed to change their initial ratings ex post, that is, after they observe the actual ICO performance. To avoid any bias resulting from this possibility, I only consider those ratings from before the ICO was launched. I summarize the empirical predictions of the three indicators on proxies for ICO success (specifically, first-day returns, the probability of positive first-day returns, ICO proceeds, nominal first-day returns, and time to market) and failure (specifically, delisting and project failure) in Table 2.

**Table 2:** Empirical predictions: Proxies for project quality.

		(a) Management Team	(b) Vision	(c) ICO Profile
1	First-Day Returns	+	?	+
2	Probability of First-Day Returns > 0	+	?	+
3	ICO Gross Proceeds	+	?	+
4	Nominal First-Day Returns	+	?	+
5	Time-To-Market	-	?	-
6	Delisting	-	?	-
7	Project Failure	-	?	-

The quality of the management team is at the core of principal-agent models. Absent effective corporate governance mechanisms, poor managerial quality translates directly into agency costs [55, 56]. Examples from the ICO market are as dramatic as fraudulent actions [46], but also include significant token price deterioration after the ICO because managers fail to meet self-set milestones or simply due to erroneous coding that have led, inter alia, to hacks. Moreover, studies of the determinants of entrepreneurial success show that the ability of the founders and managers are first-order determinants of project growth and performance (see, for a survey, Da Rin, Hellmann, and Puri [57]). Therefore, the quality of the management team should have a strong positive (negative) effect on the success (failure) of ICO projects.

Less clear is the impact of a project’s vision on its success or failure. One view is that the better the vision, the higher the returns on average [58]. A contrary view suggests that highly visionary projects are more likely to fail because disruptive innovations are more difficult to implement [59]. Given the uncertain nature of the entire cryptocurrency industry up to this point in time, the negative relationship between vision and success might be even more pronounced in the ICO market. Therefore, I acknowledge that theoretical predictions of the effect of a project’s vision are ambiguous.

The ICO profile should have a positive (negative) effect on the success (failure) of cryptocurrency projects. A number of IPO studies show that window-dressing is positively related to the funds raised in IPOs (e.g., [60]). Nevertheless, to the extent that a professional ICO profile can be created with relatively little effort and a highly sophisticated profile can not fully disguise a weak management team or a worthless vision, the effect of the ICO profile is likely to be economically less substantial. For an overview, I summarize these predictions in Table 2. Further predictions related to specific determinants of the dependent variables are discussed in each section.

## III. Data, method, and initial results

The sample consists of cryptocurrency projects that started their ICOs between August 2015 and April 2018. The information on the projects and the ICOs comes from *icobench.com* and is matched with historical pricing data from *coinmarketcap.com*. Both sources are considered to administer the most comprehensive and reliable databases. However, Lyandres, Palazzo, and Rabetti [16] stress the issue of inconsistent data across different ICO aggregators. Therefore, I verify the project data with data from *icoalert.com* and validate data entries by hand, whenever they are inconsistent. I supplement the data with hand-collected information from the projects’ websites, the ICOs’ white papers (‘the ICO prospectus’), and the LinkedIn profiles of the management team members. The data collection method complied with the terms and conditions of these websites. The final data set consists of 2,131 ICOs. However, due to the different data sources and the fact that some ICOs are not publicly listed yet, the available number of observations differs along various dimensions. Specifically, each model is based on all observations for which information of all considered covariates are available. Variable definitions are shown in Table 3.

**Table 3:** Variable definitions.

Variable	Description	Sources
First-Day Raw Return	The difference of the first-day closing price and the first-day opening price over the first-day opening price. The exact formula is shown in Eq 1.	Coinmarketcap
First-Day Abnormal Return (EW)	The excess return of the coin on its first trading day, computed by adjusting the First-Day Raw Return by the equally-weighted market benchmark. The equally-weighted index is constructed based on all cryptocurrencies with available price data. The exact formula is shown in Eq 2.	Coinmarketcap
First-Day Abnormal Return (VW)	The excess return of the coin on its first trading day, computed by adjusting the First-Day Raw Return by the value-weighted market benchmark. The value-weighted index is constructed based on all cryptocurrencies with available price data and uses the market capitalization as weight. The exact formula is shown in Eq 3.	Coinmarketcap
Positive First-Day Raw Return	A dummy variable equal to one if the First-Day Raw Return is greater than zero, and zero otherwise.	Coinmarketcap
Positive First-Day Abnormal Return (EW)	A dummy variable equal to one if the First-Day Abnormal Return (EW) is greater than zero, and zero otherwise.	Coinmarketcap
Positive First-Day Abnormal Return (EW)	A dummy variable equal to one if the First-Day Abnormal Return (EW) is greater than zero, and zero otherwise.	Coinmarketcap
ICO Gross Proceeds	The total funding amount raised through the ICO in ’000s USD.	ICObench
Nominal First-Day Returns	Calculated as the product of ICO Gross Proceeds and First-Day Raw Returns in ’000s USD.	Coinmarketcap
Time-To-Market	The difference in days between the ICO start and the date the project was founded.	Project websites, LinkedIn, ICObench
Time-To-Listing	The difference in days between the ICO end and the date the project was listed on a token exchange platform.	Project websites, LinkedIn, Coinmarketcap
Delisting	A dummy variable equal to one if a listed project was delisted at one or more token exchange platforms, and zero otherwise.	ICObench, Coinmarketcap
Project Failure	A dummy variable equal to one if the project was delisted at every token exchange platform, and zero otherwise.	ICObench, Coinmarketcap
Management Team	Based on surveys among cryptocurrency experts. Some ICOs are rated by as much as 84 experts. The rating takes into account the quality of the management team and the experience of external consultants advising the project. The rating’s scale ranges from 0 (weak) to 5 (strong). Only ratings prior to the ICO launch are considered.	ICObench
Vision	Based on surveys among cryptocurrency experts. Some ICOs are rated by as much as 84 experts. The rating takes into account the vision of the project. The rating’s scale ranges from 0 (weak) to 5 (strong). Only ratings prior to the ICO launch are considered.	ICObench
ICO Profile	Based on surveys among cryptocurrency experts. Some ICOs are rated by as much as 84 experts. The rating takes into account the professionality of the ICO profile. The rating’s scale ranges from 0 (weak) to 5 (strong). Only ratings prior to the ICO launch are considered.	ICObench
CEO Legacy	A dummy variable equal to one if the CEO was involved in another cryptocurrency project, and zero otherwise.	LinkedIn
Team Size	The number of the project’s team members excluding advisors.	ICObench
ERC20	A dummy variable equal to one if the ICO tokens were created under the ERC20 standard, and zero otherwise. The ERC20 is a technical standard that contains a list of rules for developers creating smart contracts on the Ethereum blockchain.	ICObench
Legal Tender	A dummy variable equal to one if the project accepted fiat currencies during the ICO, and zero otherwise.	ICObench
Major Cryptocurrencies	A dummy variable equal to one if the project accepted only major cryptocurrencies (Bitcoin, Ethereum, Litecoin) during the ICO, and zero otherwise.	ICObench
Pre-ICO	A dummy variable equal to one if a Pre-ICO took place prior to the actual ICO, and zero otherwise.	ICObench
ICO Duration	The duration of the ICO in days.	ICObench
Market Sentiment	The buy-and-hold return on the value-weighted index over the ICO duration.	Coinmarketcap
Total Country Restrictions	The number of countries that were excluded from the ICO.	ICObench
U.S. Restriction	A dummy variable equal to one if U.S. investors were not admitted to take part in the ICO, and zero otherwise.	ICObench
KYC/Whitelist	A dummy variable equal to one if the project used a Know-Your-Customer (KYC) process or a whitelist during the ICO.	ICObench

Following the IPO literature (e.g., [48], [51], [61]), three return measures are calculated: Raw returns, equally-weighted, and value-weighted abnormal returns. For each ICO firm *i*, raw returns are defined as the return on the first trading day (first closing price, *P*_*i*,1_, minus first opening price over first opening price, *P*_*i*,0_):

$$\begin{array}{c} R_{i}=\frac{P_{i,1}-P_{i,0}}{P_{i,0}} \end{array}$$ (1)

To account for spurious market movements on the first days of trading of the sample firms, I also compute abnormal returns employing standard event-study methodology (e.g., [62], [63]). Specifically, for both measures of abnormal returns, the market-adjusted model is employed [62], where raw returns are corrected by the return on an equally-weighted and a value-weighted market benchmark. For the market benchmark, I use all cryptocurrencies listed on *Coinmarketcap*. For the equally-weighted abnormal return of each ICO firm *i* (*EWAR*_*i*_), the first-day return of firm *i*, *R*_*i*_, is corrected by the equally-weighted average return of all other listed cryptocurrencies, *j* = 1, …, *n*, on the first trading day, *t*, of ICO firm *i*’s token:

$$\begin{array}{c} EWAR_{i}=R_{i}-\frac{1}{n}\sum_{j=1,\;j\neq i}^{n}\frac{P_{j,t}-P_{j,t-1}}{P_{j,t-1}} \end{array}$$ (2)

Similarly, the value-weighted abnormal return for each ICO firm *i* (*VWAR*_*i*_) is computed as the difference between ICO firm *i*’s first-day return, *R*_*i*_, and an average market return on the first-trading day *t* of token *i* weighted by the market capitalization, *MC*_*j*,*t*_, of every other listed cryptocurrency token *j* = 1, …, *n*.

$$\begin{array}{c} VWAR_{i}=R_{i}-\sum_{j=1,j\neq i}^{n}[\frac{MC_{j,t}}{\sum_{j=1}^{n}MC_{j,t}}\cdot \frac{P_{j,t}-P_{j,t-1}}{P_{j,t-1}}] \end{array}$$ (3)

For details about the application of standard event-study methodology (cf., [62], [63]) to ICO returns and an in-depth discussion of the return distribution, see Momtaz [15].

Summary statistics of first-day returns, gross ICO proceeds, and nominal first-day returns are presented in Table 4. The mean raw return of 0.082 is statistically different from zero at the 1 percent significance level. The median raw return is clearly lower (0.026), suggesting that the distribution is positively skewed. Raw returns at the 25th percentile are negative (-0.045), while they are positive (0.19) at the 75th percentile. The abnormal returns are of similar magnitude for the equally-weighed (0.068) and the value-weighted market benchmark (0.076). Although not tabulated, all estimates are statistically highly significant.

**Table 4:** First-day returns, gross proceeds, and nominal first-day returns.

	N	Mean	St. Dev.	Q1	Median	Q3
First-Day Raw Returns	302	0.082	0.256	-0.045	0.026	0.190
First-Day Abnormal Returns (EW)	302	0.068	0.314	-0.105	0.034	0.243
First-Day Abnormal Returns (VW)	302	0.076	0.274	-0.088	0.033	0.205
Positive First-Day Raw Return (dummy)	302	0.605	0.490	0	1	1
Positive First-Day Abn. Ret. (EW) (dummy)	302	0.543	0.499	0	1	1
Positive First-Day Abn. Ret. (VW) (dummy)	302	0.574	0.495	0	1	1
ICO Gross Proceeds, in ’000s USD	501	15,057	28,057	1,546	5,800	18,000
Nominal First-Day Returns, in ’000s USD	302	1,082	7,040	-82	0	905

**Fig 2:**
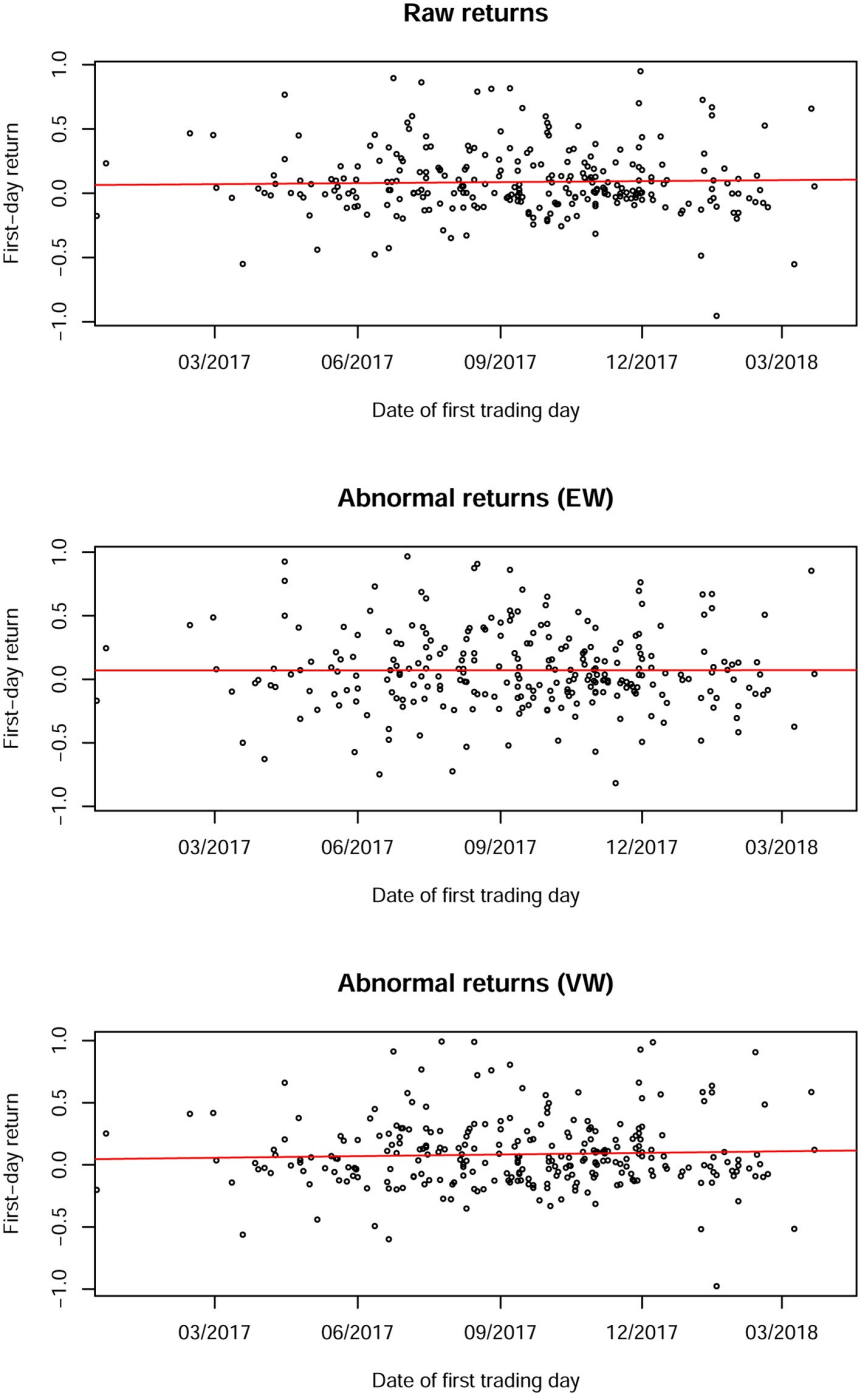
Raw returns, equally- and value-weighted abnormal returns since January 2017. The line in each graph comes from a regression of first-day returns on the date. The lines suggest that there is no linear time trend in first-day returns. Return data is available for 302 ICOs. The equally- and value-weighted returns are adjusted by an index using price data of all cryptocurrencies available from *coinmarketcap*.

Given the soaring increase in market activity over the sample period, it is necessary to check whether this affected the first-day returns over time. For that purpose, Fig 2 plots raw returns as well as equally- and value-weighted abnormal returns over time. The graphs are truncated on the left hand side due to the relatively small amount of ICOs before 2017. The regression lines do not indicate a time trend in the average first-day returns.

Table 4 presents the distribution of projects that experience positive first-day returns. Only about 54.3–60.5% of all projects have positive first-day returns. Table 4 also reports gross ICO proceeds and nominal first-day returns (both in ’000s $), with the latter measured as the product of gross ICO proceeds and raw returns. Hence, nominal first-day returns may partly reflect the financial incentives in nominal terms provided to investors by ICO firms to ensure a liquid secondary market for their tokens. The average project raises $15 millions and generates $1 million in nominal first-day returns.

**Fig 3:**
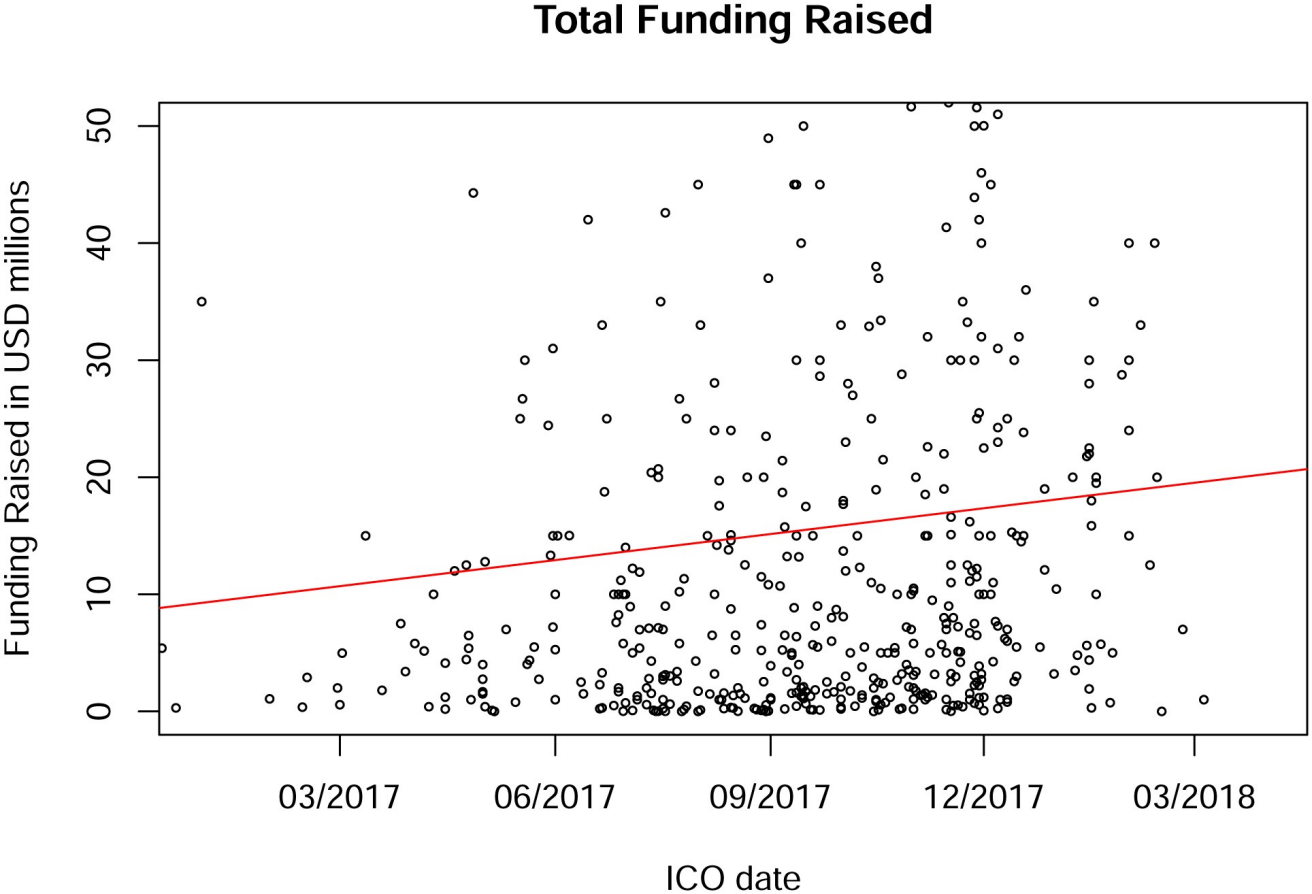
Total ICO proceeds since January 2017. The line comes from a regression of ICO proceeds on the date and indicates an increasing trend of about USD 13,000 per day. Data on gross proceeds is available for 501 ICOs. The graph is truncated at USD 50 million.

Anecdotal evidence suggests that gross ICO proceeds have increased dramatically over time. Fig 3 confirms this. The regression line indicates that the average gross proceeds per ICO increase by more than $13,000 per day.

Table 5 presents time-to-market indicators and delisting data. The average project starts its ICO 20 months (598 days) after its founding date, whereas half of all ICOs take place after only 10 months (312 days). The founding dates come from the ICO firms’ own reports on their LinkedIn websites. Untabulated results indicate that very recent ICOs dominate the subsample of very early ICOs. Once a project has raised funds, it takes, on average (median), 93 (42) days from the end of the ICO until the first token exchange listing.

**Table 5:** Time-to-market and probability of failure.

	N	Mean	St. Dev.	Q1	Median	Q3
Panel A: Indicators of Project Efficiency						
Time-To-Market, in days	875	598	1,596	173	312	672
Time-To-Listing, in days	305	93	209	22	42	71
Panel B: Indicators of Project Failure						
Delisting	495	0.210	0.408	0	0	0
Project Death	495	0.129	0.336	0	0	0

Because the success of cryptocurrencies depends primarily on its usage, a frequent feature is that they seek listing at as many exchanges as possible. I gather token data from the largest 26 token exchanges. Panel B of Table 3 shows that 21% of all projects have been delisted at least at some exchange, while 12.9% were delisted at all exchanges, suggesting that these projects collapsed and resulted in full losses for their investors. Although there were more than 200 token exchanges during the sample period, a delisting from one of the 26 major platforms leads effectively to full losses for investors. The claim is supported by evidence showing that delisting announcements on major platforms caused affected token prices to plummet to zero. Prominent examples include the delistings of tokens from *Binance* such as BCN, CHAT, ICN, and TRIG.

Table 6 summarizes the sample characteristics of the remaining dimensions and, in particular, for the dimensions of project quality. The quality of management team, vision, and ICO profile are based on independent expert ratings on the *ICObench* platform. Some ICOs received expert evaluations from as many as 84 analysts. While experts are allowed to revise their assessments subsequently, an important feature of my study is that only ex ante ratings are considered, which should effectively eliminate any look-back bias. The scale on all three dimensions ranges from 1 (weak) to five (strong). As an initial observation, the average rating for ICO profile clearly exceeds the other two dimensions, suggesting ‘window-dressing’ to a notable extent that investors might see through.

**Table 6:** Project quality and ico and project characteristics.

	N	Mean	St. Dev.	Q1	Median	Q3
Panel A: Project Quality						
Management Team	2,131	1.917	1.879	0.000	2.000	3.800
Vision	2,131	1.943	1.894	0.000	2.000	3.875
ICO Profile	2,131	3.166	1.027	2.400	3.100	4.000
Panel B: ICO and Project Characteristics						
Team Size	2,131	10.554	7.808	5	9	15
CEO Legacy	2,131	0.233	0.423	0	0	1
Pre-ICO	2,131	0.439	0.496	0	0	1
ERC20	2,131	0.673	0.469	0	1	1
Legal Tender	2,131	0.097	0.296	0	0	0
Major Cryptocurrency	2,131	0.817	0.387	1	1	1
U.S. Restriction	2,131	0.138	0.345	0	0	0
KYC/Whitelist	2,131	0.258	0.437	0	0	1

## IV. Determinants of first-day returns

This section examines the determinants of first-day returns and the probability of positive first-day returns. To that end, I regress the three measures of first-day returns on the explanatory dimensions of project quality (management team, vision, and ICO profile) and a vector of controls. Because first-day returns appears to converge to its largely time-invariant average over the sample period, the standard errors are adjusted for heteroskedasticity and clustered by quarter-years.

The regression results are shown in Table 7. Models (1) regresses raw returns, (2) uses abnormal returns corrected by the equally-weighted benchmark, and (3) uses abnormal returns corrected by the value-weighted benchmark. The parameter estimates are fairly stable across model specifications. Model (1) suggest that the quality of the management team has a significantly positive marginal effect on first-day returns, whereas vision is significantly negatively related to first-day returns. ICO profile is positively but insignificantly related to the dependent variable. Among the control variables, there is a statistically significant effect when a project uses the technical standard *ERC20* that requires projects to implement a predefined set of rules when creating their tokens. The marginal effect of ERC20 explains, ceteris paribus, 10.6% of the observed first-day returns. Moreover, the general market sentiment is also significantly positively related to first-day returns. Further, models (2) and (3) exhibit a negative coefficient for CEO legacy, which is consistent with the notion that the stigma of previous failure becomes a self-fulfilling prophecy in future projects [64]. Finally, note that the R-squared amounts to 6.66% and is thus comparable to those in widely-cited studies in the IPO underpricing literature [32].

**Table 7:** The determinants of first-day returns.

	Raw Ret.	Abn. Ret. (EW)	Abn. Ret. (VW)
	(1)	(2)	(3)
Management Team	0.0526***	0.0675	0.0573***
	(0.0092)	(0.0451)	(0.0126)
Vision	-0.0567***	-0.0758*	-0.0557**
	(0.0093)	(0.0436)	(0.0233)
ICO Profile	0.0035	-0.0159	-0.0037
	(0.0224)	(0.0284)	(0.0230)
ERC20	0.1061**	0.1064*	0.0962*
	(0.0413)	(0.0625)	(0.0509)
CEO Legacy	-0.0797	-0.0835*	-0.0633*
	(0.0507)	(0.0457)	(0.0375)
Market Sentiment	0.00001*	0.000001	0.00001**
	(0.000003)	(0.000005)	(0.000003)
ICO Gross Proceeds	-0.0000	-0.0000	0.0000
	(0.0000)	(0.0000)	(0.0000)
Constant	0.0032	0.0825	-0.0142
	(0.0641)	(0.0826)	(0.0660)
No. Observations	224	224	224
*R*^2^	6.66%	4.3%	6.04%
p-value	0.037	0.133	0.059

This table provides the regression results for the determinants of the first-day returns. First-day return data are available for 302 ICOs, however, I loose some observations due to lacking information for the determinants. The dependent variable in models (1), (2), and (3) are First-Day Raw Returns, equally-weighted Abnormal Returns, and value-weighted Abnormal Returns, respectively. Model (2) has a relatively poor fit because the equally-weighted index introduces a significant amount of noise. The independent variables are explained in Table 3. Standard errors reported in parentheses below the coefficients are adjusted for heteroskedasticity and clustered by time (quarter-years).

***, **, and * stand for statistical significance at the 1%, 5%, and 10% level, respectively.

Table 8 presents results from linear probability models, estimating the probability that the first-day return of a given ICO is greater than zero. Here, models (1), (2), and (3) use dummy variables equal to one if the raw return, the equally-weighted abnormal return (EWAR), or the value-weighted abnormal return (VWAR), respectively, is strictly positive. Again, the standard errors are adjusted for heteroskedasticity and clustered by quarter-years.

**Table 8:** Probability of positive first-day returns.

	Raw Ret. > 0	Abn. Ret. (EW) > 0	Abn. Ret. (VW) > 0
	(1)	(2)	(3)
Management Team	0.0860***	0.0920	0.1347**
	(0.0284)	(0.0693)	(0.0662)
Vision	-0.1005***	-0.0987	-0.1418**
	(0.0316)	(0.0670)	(0.0639)
ICO Profile	-0.0253	-0.0735*	-0.0268
	(0.0416)	(0.0437)	(0.0417)
ERC20	0.1285*	0.1343	0.1249
	(0.0690)	(0.0961)	(0.0922)
CEO Legacy	-0.1236**	-0.0486	-0.0866
	(0.0572)	(0.0703)	(0.0529)
Market Sentiment	0.00001*	0.00001*	0.00002***
	(0.00001)	(0.000004)	(0.00001)
ICO Gross Proceeds	-0.0000	-0.0000*	0.0000
	(0.0000)	(0.0000)	(0.0000)
Constant	0.6035***	0.7065***	0.4879***
	(0.1192)	(0.1270)	(0.1196)
No. Observations	224	224	224
*R*^2^	4.59%	3.96%	6.86%
p-value	0.068	0.114	0.030

This table provides the regression results for the determinants of the probability of positive first-day returns. First-day return data are available for 302 ICOs, however, I loose some observations due to lacking information for the determinants. The dependent variable in models (1), (2), and (3) are indicator variables equal to one if First-Day Raw Returns > 0, equally-weighted Abnormal Returns> 0, and value-weighted Abnormal Returns> 0, respectively. Model (2) has a relatively poor fit because the equally-weighted index introduces a significant amount of noise. The independent variables are explained in Table 3. Standard errors reported in parentheses below the coefficients are adjusted for heteroskedasticity and clustered by time (quarter-years).

***, **, and * stand for statistical significance at the 1%, 5%, and 10% level, respectively.

The regression results are consistent with the main implications in Table 7. In terms of standard deviations, a one-standard deviation increase in management quality increases the probability of positive first-day returns by 25.32% in Model (3). On the other hand, a one standard deviation increase in the project’s vision reduces the probability of positive first-day returns by 28.86%.

Overall, the results presented in this section support the hypothesis that management team quality is positively related to first-day returns, while project vision has a negative effect. While the latter finding may look surprising on the surface, the analysis below shows that the discount on visionary projects can be explained by a higher probability of failure.

## V. Gross proceeds and nominal first-day returns

To what extent do project quality and investor uncertainty about project quality affect the amount of gross proceeds and nominal first-day returns in ICOs? The results are shown in Table 9. The dependent variables are total gross proceeds in ’000s $ in models (1) and (2) and nominal first-day returns in ’000s $ in models (3) and (4). Nominal first-day returns are measured as the product of first-day raw returns and the amount of gross proceeds. To proxy for investor uncertainty about project quality, I introduce a new set of explanatory variables. The uncertainty about project quality is measured as the variance in analyst opinions in the three dimensions: management team, vision, and ICO profile. A high value on these dimensions indicates that there is much uncertainty in the market about project quality prior to the ICO.

**Table 9:** Analysis of funding amount and nominal first-day returns (in ’000s USD).

	Total Funding		Nominal First-Day Returns	
	(1)	(2)	(3)	(4)
Management Team	9,686***		894	
	(3,158)		(1,024)	
Vision	-7,900**		-939*	
	(3,136)		(528)	
ICO Profile	2,375*		648	
	(1,210)		(573)	
Uncertainty about Management Team		2,935***		476**
		(1,074)		(210)
Uncertainty about Vision		-3,098**		-364***
		(1,240)		(92)
Uncertainty about ICO Profile		1,225		21
		(1,015)		(262)
Pre-ICO	-7,110*	-3,607		
	(3,938)	(3,879)		
ERC20	1,345	3,647**		
	(4,833)	(1,408)		
Legal Tender	10,587**	14,130***		
	(5,293)	(4,658)		
Major Cryptocurrency	3,712	4,058		
	(5,068)	(5,169)		
Market Sentiment	2,003**	2,400**		
	(1,210)	(1,013)		
ICO Duration	-196**	-193***		
	(82)	(46)		
U.S. Restriction	-1,637	-10,746**		
	(18,138)	(4,509)		
Total Country Restrictions			1,013***	759***
			(194)	(267)
KYC/Whitelist			-5,317***	-4,672***
			(968)	(781)
ICO Gross Proceeds			0.0001**	0.0001**
			(0.00003)	(0.00003)
Constant	-3,065	3,777	-1,796	-5
	(7,842)	(6,146)	(1,846)	(757)
No. Observations	132	132	243	243
*R*^2^	18.72%	14.52%	6.71%	6.36%
p-value	0.004	0.033	0.011	0.016

This table provides the regression results for the determinants of ICO Gross Proceeds and Nominal First-Day Returns. Data on ICO Gross Proceeds and Nominal First-Day Returns are available for 501 and 302 observations, respectively. However, I loose some observations due to lacking information for the determinants. The dependent variable in models (1) and (2) is ICO Gross Proceeds in ’000s USD. The dependent variable in models (3) and (4) is Nominal First-Day Returns in ’000s USD. The independent variables are explained in Table 3. Standard errors reported in parentheses below the coefficients are adjusted for heteroskedasticity and clustered by time (quarter-years).

***, **, and * stand for statistical significance at the 1%, 5%, and 10% level, respectively.

The results support my predictions. In model (1), the coefficients on quality of the management team and the ICO profile are positive, while there is a negative coefficient for vision. However, only the parameter estimate for ICO profile is statistically significant, suggesting that window-dressing pays off. In terms of standard deviations, a one standard deviation improvement in ICO Profile, ceteris paribus, results in $2.44 million higher gross proceeds.

The control variables shed more light on the determinants of ICO gross proceeds and are consistent with the expected effects. In particular, (i) the existence of a Pre-ICO reduces the total funding amount raised in the actual ICO by $7.11 million, (ii) projects accepting legal tender raise, on average, $10.586 million more as it reduces the investors’ entry barriers into the new market, (iii) the market sentiment during the ICO period as measured by the development of the Bitcoin price is significantly positively related to gross proceeds, and (iv) gross proceeds decrease in the duration of the ICO as longer fundraising periods likely indicate the project is having trouble raising the desired amount which is a negative signal to potential investors.

Looking at uncertainty about project quality in model (2), the variance in the analysts’ opinions about the quality of the management team is associated with a positive effect on gross proceeds, while uncertainty about the project’s vision has a significantly negative effect. Uncertainty about the ICO profile is insignificantly positively related to gross proceeds. In addition to the effects of the control variables documented for model (1), the results in model (2) further suggest that using the technical standard ERC20 and the CEO having a crypto-legacy are positively related to gross proceeds in ICOs.

Turning to the determinants of nominal first-day returns (in ’000s $), the results in model (3) suggest that only vision has a significantly negative effect on nominal first-day returns. Interestingly, the uncertainty about both the quality of the management team and the vision in model (4) significantly affect nominal first-day returns. The significantly positive coefficient on the uncertainty about management team quality suggests that teams with varying quality perceptions among investors have to offer higher financial incentives to acquire the desired amount of total funding.

The other variables also provide important insights into the determinants of nominal first-day returns. First, projects that restrict certain countries (mostly the U.S. and China) generate higher nominal first-day returns. An additional restriction is associated with an increase by $0.76 million. This finding is consistent with the notion that reducing the set of potential investors requires higher incentives for the remaining to raise the desired funding amount. Second, there is a negative effect on nominal first-day returns if the project raises funds during the ICO using a KYC (Know-Your-Customer) process or a white list. The coefficient indicates a reduction of nominal first-day returns in the amount of $4.67 million. The finding can be interpreted in the way that verified identities reduce the threat of potential liabilities under anti-money laundering regulations. Hence, lacking a KYC process leads investors to demand higher financial incentives for bearing the extra risk of potential lawsuits. Third, the analysis suggests a statistically and economically significant size effect. An additional dollar of funding raised is associated with additional $0.065 of nominal first-day returns. This finding is also consistent with the IPO literature.

## VI. Time-to-market and market exit

Important additional dimensions of the success of ICOs concern the timing of market entry and the probability of failure. I proxy for market entry by the time (in days) it takes a project to start its ICO after its initiation. The probability of failure is measured, first, by the probability that a project token gets delisted at least at one major token exchange, and, second, by the probability that it gets delisted on all major exchanges, which is evidence of total project failure. Table 10 reports regression results for these three variables. The results reported in this section are robust to the alternative model specification following a frailty approach, for details see Momtaz [65].

**Table 10:** Time-to-market and the probability of delisting of cryptocurrencies.

	Time-To-Market	Delisting	Project Death
	(1)	(2)	(3)
Management Team	-19.9792	-0.1023**	-0.1053**
	(185.2532)	(0.0503)	(0.0457)
Vision	34.0592	0.1195**	0.1133 ***
	(184.9778)	(0.0466)	(0.0424)
ICO Profile	-104.1703**	-0.0038	-0.0261*
	(48.2517)	(0.0365)	(0.0156)
Uncertainty about Management Team	51.7313		
	(78.2695)		
Uncertainty about Vision	-39.8212		
	(75.1265)		
Uncertainty about ICO Profile	13.8994**		
	(6.9161)		
Team Size	6.5238		
	(8.5030)		
CEO Legacy	-22.9099	0.0282	0.0748
	(111.6980)	(0.0580)	(0.0527)
Legal Tender	388.8109**	-0.0418*	-0.0205*
	(171.7112)	(0.0234)	(0.0113)
Total Country Restrictions		-0.0053**	-0.0060 ***
		(0.0022)	(0.0020)
KYC/Whitelist	211.1213 ***	0.0065	0.0575
	(39.5117)	(0.0735)	(0.0668)
Constant	702.4376 ***	0.1041	0.0842
	(240.6550)	(0.1153)	(0.1048)
No. Observations	875	495	495
*R*^2^	14.60%	13.67%	12.03%
p-value	0.049	0.039	0.084

This table provides the regression results for the determinants of Time-To-Market and Project Failure. There are 875 observations for which the founding date and the ICO date are known, and 495 ICOs whose listing status is known. The dependent variable in model (1) is Time-To-Market in days. The dependent variable in models (2) and (3) is Delisting and Project Death, respectively. All variables are explained in Table 3. Standard errors reported in parentheses below the coefficients are adjusted for heteroskedasticity and clustered by time (quarter-years).

***, **, and * stand for statistical significance at the 1%, 5%, and 10% level, respectively.

Regarding the indicators of project quality, a one-notch improvement in the attractiveness of the ICO profile reduces the time-to-market by statistically significant 104 days. However, a one-unit increase in the uncertainty about the ICO profile increases time-to-market by 14 days. Furthermore, a major determinant of time-to-market is whether the ICO uses a KYC process or a white list, which procrastinates the ICO on average by 211 days. In a similar vein, if a legal tender is accepted during the ICO, the project goes public on average 389 days later than the projects in the comparison group. The latter finding is explained by the fact that during the early days of the ICO market, cryptocurrencies were in almost every jurisdiction not considered to be an asset, hence the regulatory effort associated with the ICO were less time-consuming.

Looking at the factors influencing the probability of failure in the linear probability models (2) and (3) of Table 10, the dimensions of project quality, as estimated before and during the ICO, are fairly accurate predictors of future delistings. Model (3) indicates that a one-standard deviation increase in the quality of the management team reduces the probability of a project’s death (delisted everywhere) by about 19.8% (std. dev. * coefficient = 1.879*(-0.1023)). Similarly, a one standard deviation increase in vision persuasiveness increases the probability of project failure by about 21.5%. This result is interesting in that it shows that the promise of the vision is positively related to project failures, suggesting that highly innovative projects are less likely to succeed. Finally, model (3) indicates that the ICO profile is negatively related to project failure, with a one-standard deviation change in ICO Profile lowering the probability of delistings by 2.7%.

The other explanatory variables suggest that ICOs accepting legal tender and restricting some countries are less likely to fail. Specifically, a project accepting legal tender as a means of payment for its tokens during the ICO is associated with a lower probability of failure by 2.1%. Moreover, country restrictions during the ICO are also associated with less subsequent delistings. Per restriction, a project reduces its likelihood to fail by 0.6%, which may be explained by a reduced risk of litigation and regulatory action [56, 67].

## VII. The sensitivity of ICOs to adverse industry events

The results thus far suggest that there are, on average, substantial first-day returns in the ICO market. The goal of this section is to shed some light on the sensitivity of first-day raw returns to key industry events.

To that end, I screen the news for the entire sample period and identify the key events that had the most resounding echo in the crypto-industry. This leads to the six events described in Table 11. The events include three very prominent hacks of cryptocurrency projects and exchanges, namely the hacks of the *DAO project*, *Bitfinex* (a major exchange for project tokens), and the more recent one of *Parity Wallet*. There are also two governmental announcements that stand out. The first is the Chinese ban of raising funds through ICOs by companies or individuals on September 4, 2017, declaring ICOs an illegal activity. The second is the ban of ICOs and Bitcoin futures trading by the South Korean Financial Services Commission on December 6, 2017. Finally, the list of key events includes Facebook’s new ads policy, restricting advertisement of ICOs and cryptocurrency projects in general, stating that many of these projects are “not operating in good faith.”

**Table 11:** Overview of important adverse industry events.

Event	Date	Description
DAO Hack	Jun 17, 2016	The decentralized autonomous organization (DAO) was a form of an investor-directed venture capital fund. During the hack, about one third of the funds were stolen. The DAO token was subsequently delisted from token exchanges. The Ethereum community decided to hard-fork the Etherem blockchain to restore all stolen funds to its original contract. This entailed a paradigmatic debate about the inviolability of the blockchain and resulted in two conflicting ‘schools of thought’ (ETH and ETC).
Bitfinex Hack	Aug 2, 2016	The Bitfinex hack was the second-biggest hack of a token exchange platform, in which about 120,000 Bitcoins were stolen. In addition to the size of the hack, it revealed a critical governance issue. Because token exchange platforms were not obliged to verify its users’ identities and cryptocurrency transactions are irreversible, users had no viable instrument to be compensated for their losses. This exposed a central shortcoming of cryptocurrencies compared to conventional financial intermediaries, such as banks, that have a legal obligation and the necessary governance structures in place to trace back stolen accounts and cover the losses.
China’s Ban	Sep 4, 2017	China declared ICOs illegal activity and banned all companies and individuals from raising funds through ICOs. The regulatory action was endorsed by China’s Securities Regulatory Commission, its Insurance Regulatory Commission, and the People’s Bank of China, among others.
Parity Wallet Hack	Nov 7, 2017	The hack of popular digital wallet service provider, Parity Wallet, resulted in a loss of about USD 300 millions. It incited another discussion about a hard-fork on the Ethereum blockchain, as was the case following the DAO hack.
South Korea’s Ban	Dec 6, 2017	South Korea’s Financial Services Commission issued a ban on the trading of Bitcoin futures. While it did not ban token exchange platforms outright, it announced that ICOs will remain subject to the ban.
Facebook’s New Ads Policy	Jan 30, 2018	Facebook announced a new product advertisement policy prohibiting the promotion of ICOs on Facebook, a major marketing channel for cryptocurrency projects hitherto. The sharpness of Facebook’s statement unsettled the market: “We’ve created a new policy that prohibits ads that promote financial products and services that are frequently associated with misleading or deceptive promotional practices, such as binary options, initial coin offerings and cryptocurrency”.

**Fig 4:**
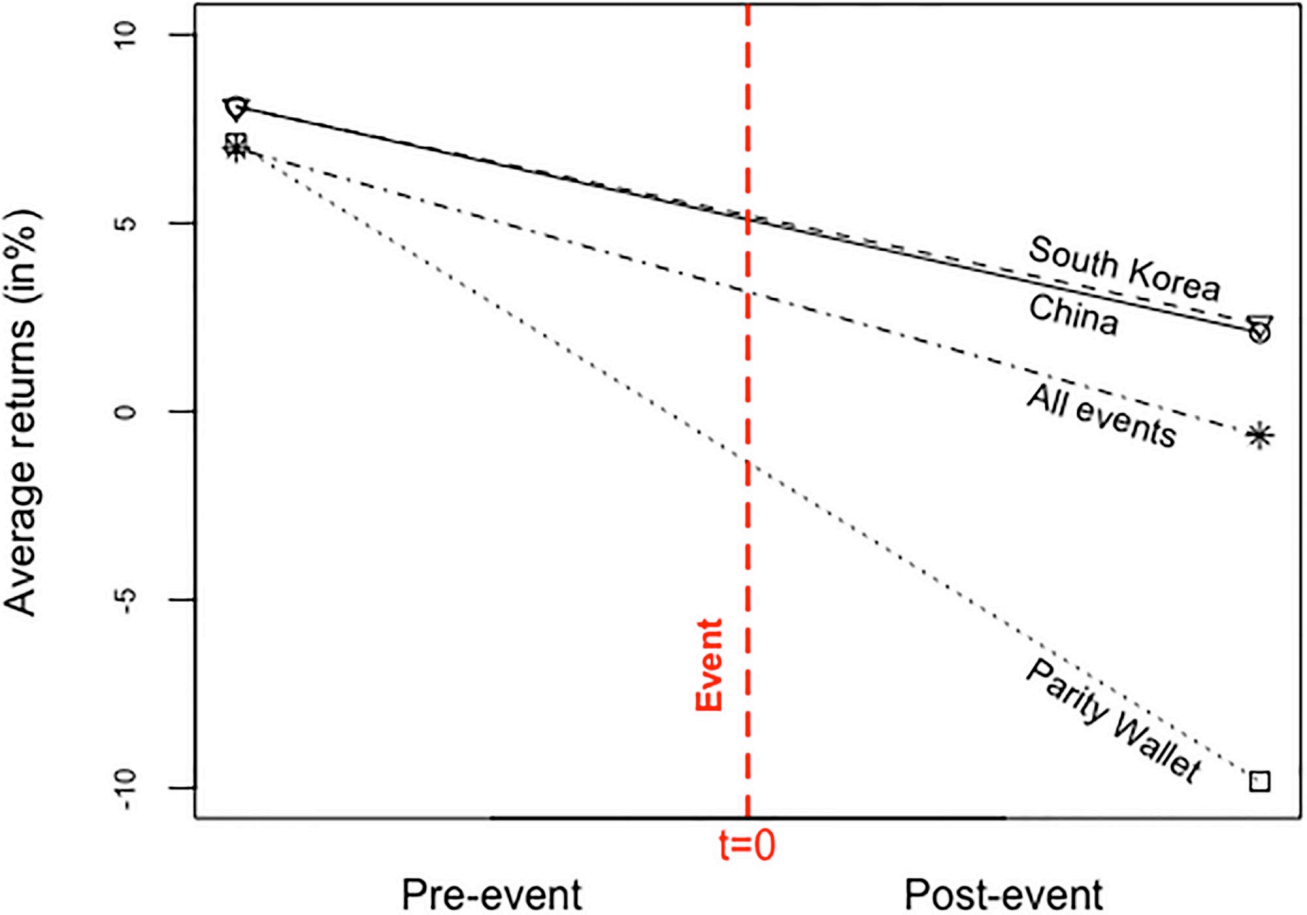
Average returns before and after adverse industry events. The figure shows average first-day returns of ICOs that took place within the month before and within the month after significant, adverse industry events (where t = 0 corresponds to the focal event). The following events are considered: China’s ban of ICOs on September 4, 2017, South Korea’s ban of ICOs on December 6, 2017, and the hack of Parity Wallet on November 7, 2017.

Graphical evidence of the impact of China’s and South Korea’s ICO bans as well as the hack of *Parity Wallet* is shown in Fig 4. In particular, the graph illustrates average first-day returns of ICOs that were listed before or after the month the focal event took place. All adverse industry events had a detrimental impact on first-day returns, although the effects’ magnitudes differ. For example, the decrease in first-day returns due to the hack of *Parity Wallet* was twice the size of the decreases due to China’s and South Korea’s regulatory bans. These effects are discussed further below, where ultivariate regression analyses are presented.

To control for potential confounding factors, a straightforward OLS regression approach is employed to analyze the market impact of the industry events. Specifically, to capture the events’ effects on first-day returns, I include binary variables in the regression models used to explain first-day returns in section IV that equal one if an ICO takes place one month after the focal event. Unreported results show that the results are robust to using shorter time windows such as two weeks.

The regression results are reported in Table 12. In model (1), the binary variable is an aggregate index of all events shown in Table 11. Models (2), (3), and (4) show the effects of specific events, namely the Parity Wallet Hack, the Chinese ban, and the South Korean ban.

**Table 12:** Sensitivity of first-day raw returns to adverse industry events.

	(1)	(2)	(3)	(4)
All Events	-0.0762***			
	(0.0165)			
Parity Wallet Hack		-0.1693*		
		(0.0898)		
China’s Ban			-0.0601***	
			(0.0223)	
South Korea’s Ban				-0.0576*
				(0.0331)
Management Team	0.0514***	0.0530***	0.0506***	0.0536***
	(0.0107)	(0.0080)	(0.0108)	(0.0101)
Vision	-0.0560***	-0.0558***	-0.0550***	-0.0581***
	(0.0071)	(0.00098)	(0.0081)	(0.0079)
ICO Profile	0.0008	0.0004	0.0050	0.0008
	(0.0224)	(0.0225)	(0.0225)	(0.0227)
ERC20	0.1108**	0.1122**	0.1065**	0.1068**
	(0.0499)	(0.0496)	(0.0495)	(0.0495)
CEO Legacy	-0.0792**	-0.0808**	-0.0809**	-0.0784**
	(0.0365)	(0.0366)	(0.0367)	(0.0367)
Market Sentiment	0.00001**	0.00001	0.00001*	0.00001*
	(0.000004)	(0.000004)	(0.000003)	(0.000004)
ICO Gross Proceeds	-0.0000	-0.0000	-0.0000	-0.0000
	(0.0000)	(0.0000)	(0.0000)	(0.0000)
Constant	0.0053	0.0121	0.0023	0.0031
	(0.0639)	(0.0643)	(0.0642)	(0.0641)
No. Observations	224	224	224	224
*R*^2^	7.46%	7.36%	6.82%	6.87%
p-value	0.033	0.036	0.054	0.052

This table provides the regression results for the sensitivity of first-day raw returns to important industry events. First-day return data are available for 302 ICOs, however, I loose some observations due to lacking information for the determinants. The dependent variable in all models is the First-Day Raw Return. The independent variables are explained in Table 3. Standard errors reported in parentheses below the coefficients are adjusted for heteroskedasticity and clustered by time (quarter-years).

***, **, and * stand for statistical significance at the 1%, 5%, and 10% level, respectively.

In model (1), the parameter estimate for the aggregate industry events variable is significantly negative. It suggests that ICOs following these events experience, on average, 7.62% lower first-day raw returns than ICOs in more optimistic times, demolishing almost all gains for first-day investors. The other parameter coefficients in model (1) are consistent with those reported for the corresponding models in Table 7. In particular, management team quality is positively related to first-day returns, while project vision has a negative effect. Also, both the use of the technical standard ERC20 and the market sentiment are significantly positively related to first-day returns.

To further shed some light on the relevance of individual events, first-day returns are regressed on binary variables for the events separately. In these models of the events’ individual effects, I also control for all other events that affected first-day returns but suppress them here as they are similar to the ones reported in the other columns. To ensure statistically meaningful results, I focus on the hack of Parity Wallet and the ICO bans by the Chinese and the South Korean governments as these three events happened during times of very high ICO activity, ensuring a sufficiently large number of observations. The hack of Parity Wallet in model (2) is associated with the highest negative effect observed among all events. The coefficient indicates that, subsequent to the hack, ICOs exhibited first-day returns that were, ceteris paribus, 16.93% lower than the average ICO in other times. This translates into first-day losses of about 8.7%. Although, events that cast doubt on the technological robustness of cryptocurrency projects unsettle the crypto-industry to the highest extent, adverse governmental announcements have also economically and statistically significant effects as the bans by Chinese and South Korean regulators exemplify. Model (2) reports a significantly negative coefficient on the binary variable for the Chinese ban of ICOs. It amounts to -6.01%, suggesting that it lessened average first-day returns (8.2%) by about three-fourths in the global market. The Korean ban had an effect of similar magnitude. ICOs following this event experienced 5.76% lower first-day returns. Again, the other variables are consistent with the ones documented in the benchmark models in Table 7, suggesting that the key determinants of ICO first-day returns are stable predictors even during adverse industry events.

In untabulated results, I find consistent results for nominal first-day returns. Specifically, the dummy used in model (1) for all adverse industry events indicates that ICOs following these events generate about $0.62 in nominal first-day returns (p-value: 2.27%). It is important to note that this is not determined by the project. Rather, nominal first-day returns following adverse industry effects have to be interpreted in the sense that event-induced industry uncertainty constrains the realization of project returns in the very short run.

Overall, the results illustrate the high level of uncertainty in the cryptocurrency industry as ICO returns are highly sensitive to adverse industry effects. In particular, the results suggest that events highlighting the technological risks of cryptocurrencies are associated with more severe market downturns than adverse regulatory announcements aiming at investor protection.

## VIII. Limitations

Because data available for research on the ICO market comes with several caveats, it is important to discuss how the limitations affect my study. Specifically, their are two threats to internal validity. First, my definition of first-day returns compares opening and closing prices for each ICO firm on its first trading day, as reported by Coinmarketcap. However, token markets are active 24/7 and the exact time a token is listed, i.e., the opening time, is not known. This implies that not all first-day returns may be calculated for the full 24-hours period. To make sure this does not introduce a systematic bias, I also computed initial returns for the first two and three days of trading, respectively. This reduces the relative difference in the time periods used to compute initial returns. Reconfirming evidence shows that the results for first-day returns do not change materially when I consider these longer periods, suggesting the regression results for first-day returns are not significantly biased.

Second, data from Coinmarketcap tracks token prices on 26 major exchange platforms. However, during the sample period, there were about 200 exchange platforms. Therefore, a delisting on a major exchange does not necessarily imply that the project has failed as it may still trade on a smaller exchange platform. In fact, a delisting does not necessarily have to be associated with poor performance; it may also reflect a strategic move on the part of the ICO firm (e.g., in order to save on maintenance and liquidity costs). Unfortunately, there is no systematic data on the activity on all exchanges nor on the reasons of delistings. Therefore, to ensure that delistings reflect project failure in my sample, I verified the reasons for each delisting manually. Indeed, among those ICOs used for the regression analyses (i.e., the ICOs with documentation of all required control variables), delistings were all associated with detrimental news about the projects.

A final limitation pertains to the external validity of the study. My sample merges ICObench and Coinmarketcap data, with an overlap of about 20% of the data. It is not clear whether the ICOs documented in both data sets are systematically different from ICOs only documented in one. Therefore, the results can only be interpreted *locally*, that is, for those ICOs covered by both sources. Further, the final sample size is reduced for three additional reasons. First, ICObench started operating in 2017. Second, the final sample considers only those ICOs for which I have access to expert ratings published before the ICO launch to avoid any look-back bias. Third, to ensure that my results are internally consistent, the final sample considers only utility tokens. This leads to a somewhat reduced sample size compared to concurrent studies such as Kostovetsky and Benedetti [14] and Howell, Niessner, and Yermack [17]. While these limitations are necessary to avoid biases of the models’ internal validity, they reduce the generalizability of the results. Therefore, the results should be interpreted locally for the final sample. Better data availability in the future may allow for research that goes beyond these limitations.

## IX. Conclusion and further research

The purpose of this paper is to document an initial set of stylized facts in the ICO market. The study has provided an empirical characterization of key ICO market outcomes such as first-day returns, gross proceeds, time-to-market, and project failure, as well as their determinants. The quality of the management team is a first-order predictor for the success of ICO projects, whereas highly visionary projects trade at a discount due to an increased probability of failure. An event study suggests that the ICO market is very sensitive to adverse industry events. Both technical hacks and adverse regulatory actions destroy substantial tokenholder value, with the former effect being more than twice as strong.

This study flags a long list of promising avenues for future research that is partly reflected in concurrent studies. One unresolved issue concerns the *longitudinal performance* of ICOs. It is not clear what fraction of ICOs survives in the long run and how their token prices evolve (see, for first long-term evidence, [16], [15], [14]). Another unresolved issue concerns the fraction of ICOs that fail before getting funded or listed, although this number might be large [17]. Current data availability does not allow to examine the determinants of *premature failure* (or fraud). Furthermore, understanding the *underlying mechanisms behind ICO market outcomes* requires further research. For example, positive initial returns are predicted by several explanations (e.g., market liquidity or hot markets), but we lack an understanding of the relevant importance of these competing mechanisms.

## Supporting information

S1 File (ZIP)
