# Supplementary figures and images for: Initial Coin Offerings
Source: PLoS One. 2020 May 21;15(5):e0233018. doi: 10.1371/journal.pone.0233018 (PMC7241798; doi:10.1371/journal.pone.0233018)

Number of ICOs since January 2017

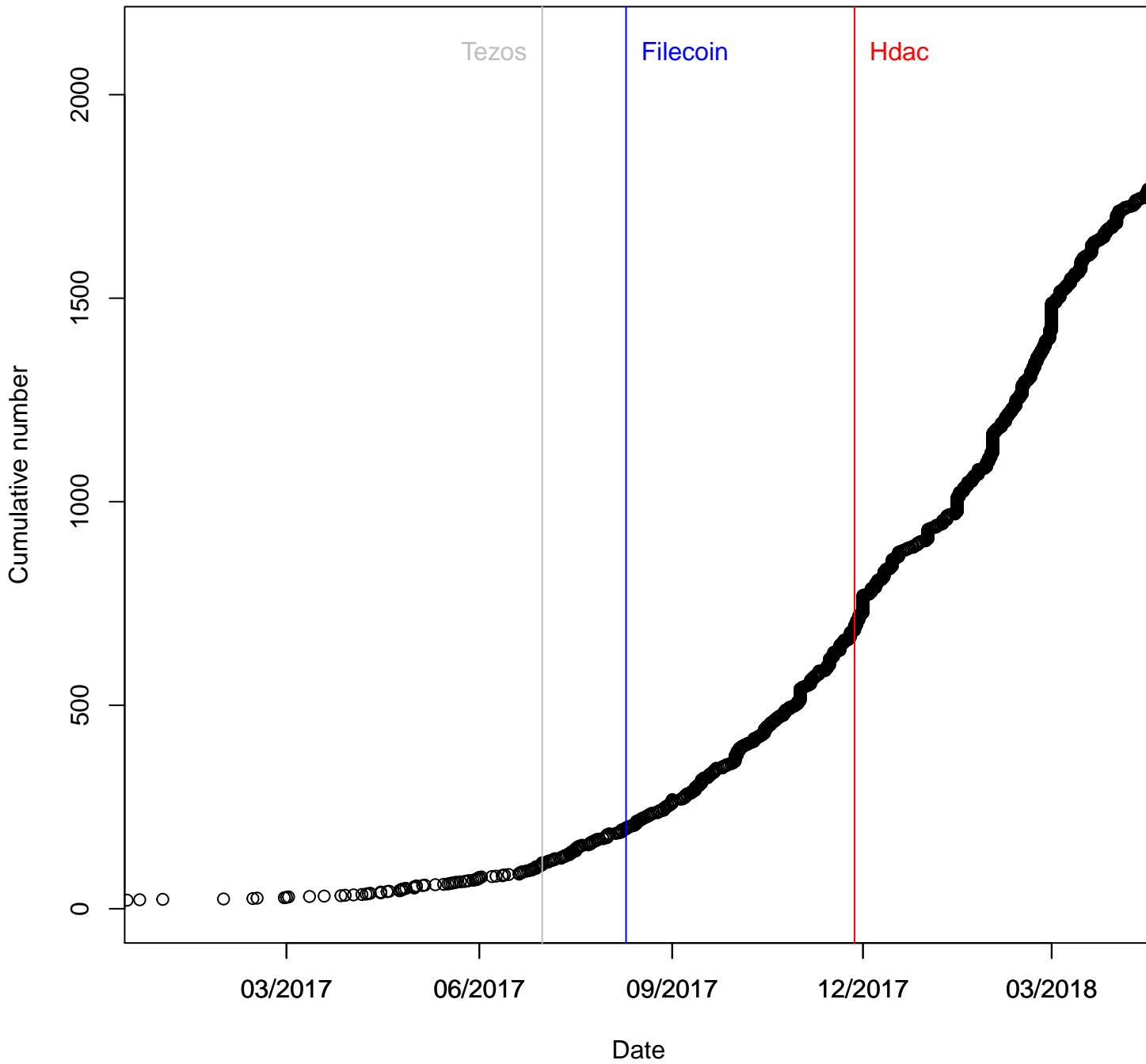

Cumulative ICO Proceeds since January 2017

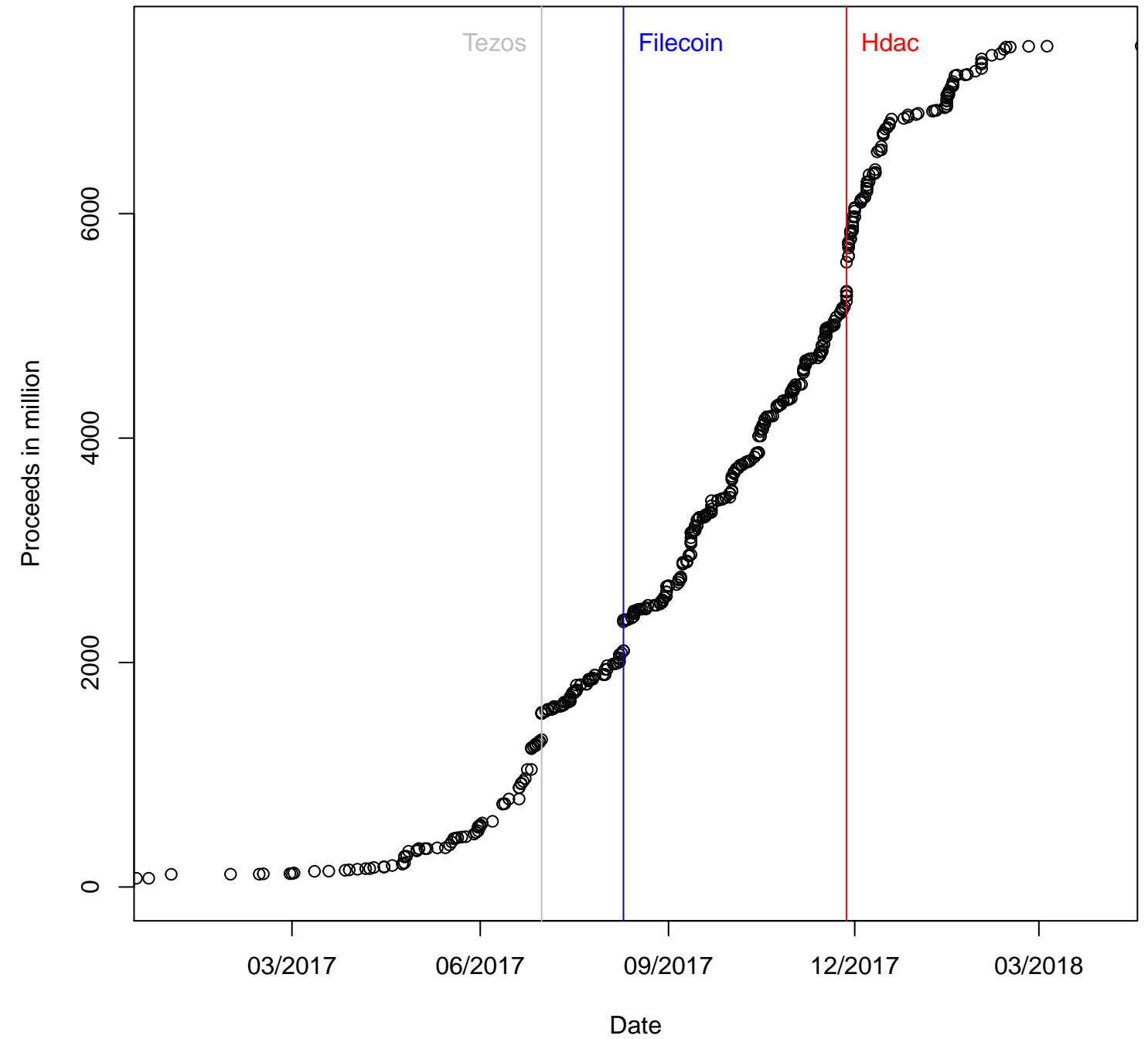

Supplement: S1 File — (ZIP) [file pone.0233018.s001.zip › Figure1.pdf]

### Raw returns

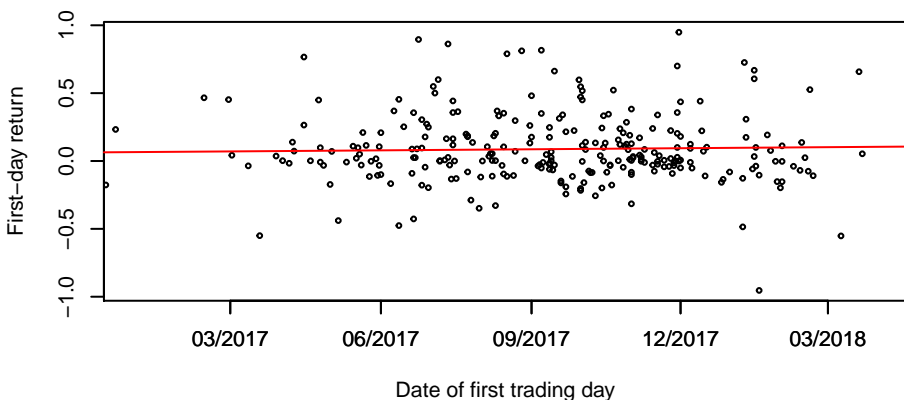

### Abnormal returns (EW)

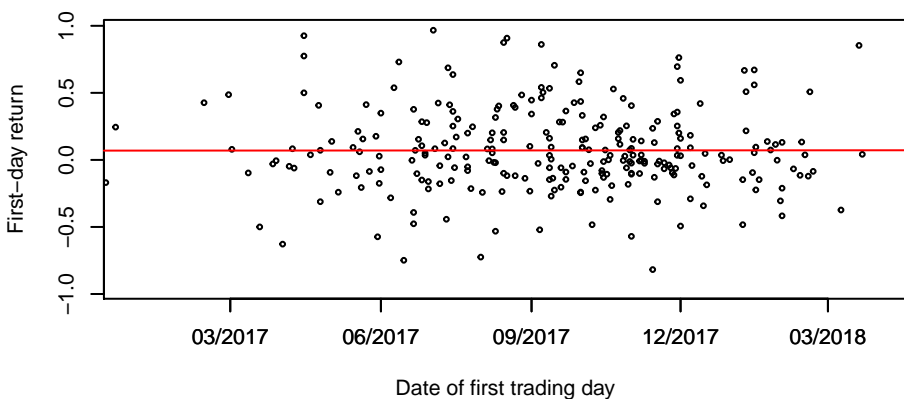

### Abnormal returns (VW)

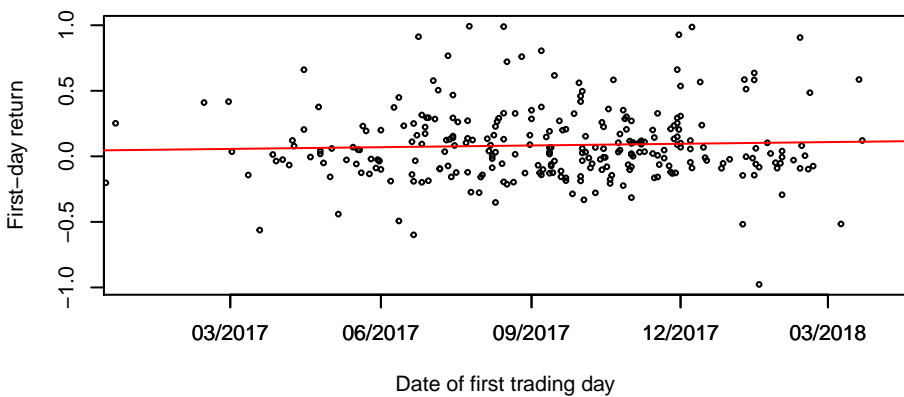

Supplement: S1 File — (ZIP) [file pone.0233018.s001.zip › Figure2.pdf]

# Total Funding Raised

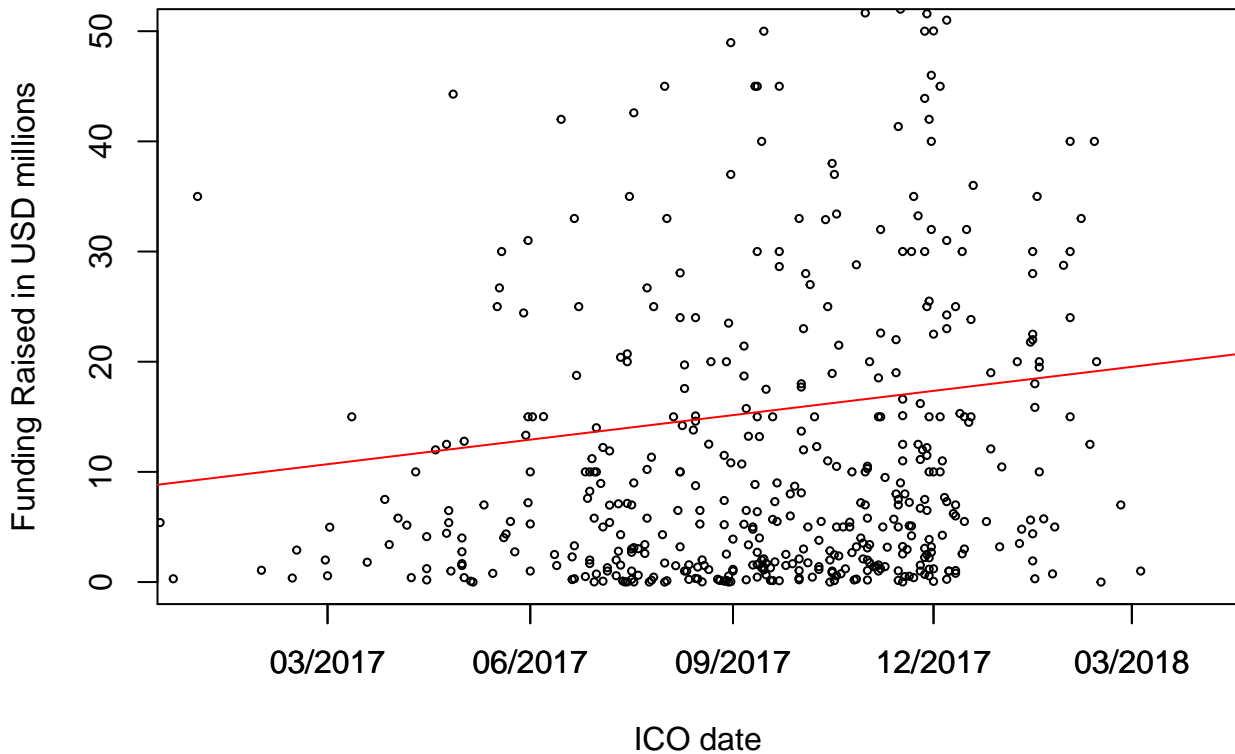

Supplement: S1 File — (ZIP) [file pone.0233018.s001.zip › Figure3.pdf]

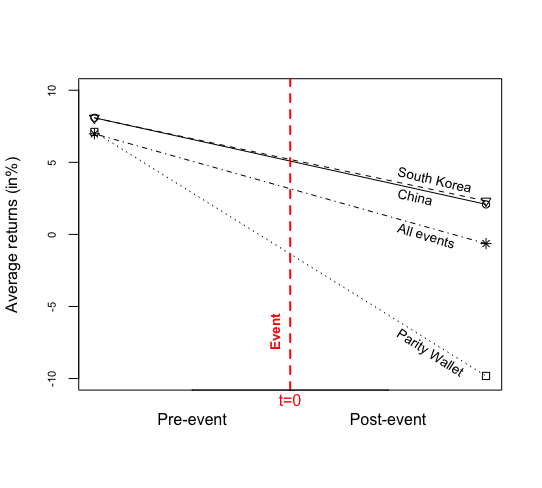

Supplement: S1 File — (ZIP) [file pone.0233018.s001.zip › GraphicalAnalyses.png]
